# Integrated Metabolomic and Transcriptomic Analysis of the Quinoa Seedling Response to High Relative Humidity Stress

**DOI:** 10.3390/biom13091352

**Published:** 2023-09-05

**Authors:** Xinyi Li, Ping Zhang, Jia Liu, Hongxin Wang, Junna Liu, Hanxue Li, Heng Xie, Qianchao Wang, Li Li, Shan Zhang, Liubin Huang, Chenghong Liu, Peng Qin

**Affiliations:** 1 College of Agronomy and Biotechnology, Yunnan Agricultural University, Kunming 650201, China2021110031@stu.ynau.edu.cn (P.Z.); 2021240157@stu.ynau.edu.cn (H.W.); 2021110026@stu.ynau.edu.cn (J.L.); 2021210172@stu.ynau.edu.cn (H.L.); 2020210159@stu.ynau.edu.cn (H.X.); 2020110028@stu.ynau.edu.cn (Q.W.); 2019210130@stu.ynau.edu.cn (L.L.);; 2 Yuxi Academy of Agricultural Science, Yuxi 653100, China; czi_jia@126.com; 3 Biotechnology Research Institute, Shanghai Academy of Agricultural Sciences, Shanghai 201106, China

**Keywords:** transcriptomics, metabolite accumulation, differentially expressed gene, high relative humidity, quinoa

## Abstract

Quinoa is of great interest because it is cold- and drought-resistant; however, little research has been performed on quinoa under high relative humidity (RH) stress. In this study, quinoa seedlings of a highly HR-resistant variety (“Dianli-439”) and a sensitive variety (“Dianli-969”) were subjected to morphological and physiological measurements and metabolome and transcriptome analyses to investigate their response to high RH stress. In total, 1060 metabolites were detected, and lipids and flavonoids were the most abundant, with 173 and 167 metabolites, respectively. In total, 13,095 differentially expressed genes were identified, and the results showed that abscisic acid, auxin, and jasmonic-acid-related genes involved in plant hormone signaling may be involved in the response of quinoa seedlings to high RH stress. The analysis of the transcription factors revealed that the AP2/ERF family may also play an important role in the response to high RH stress. We identified the possible regulatory mechanisms of the hormone signaling pathways under high RH stress. Our findings can provide a basis for the selection and identification of highly resistant quinoa varieties and the screening of the metabolite-synthesis- and gene-regulation-related mechanisms in quinoa in response to RH stress.

## 1. Introduction

Quinoa (*Chenopodium quinoa* Willd.), also known as quinoa grain, South American quinoa, and quinoa quinoa, was one of the major food crops of the ancient Inca peoples [1,2]. It has a high nutritional value and high protein content, and the high-quality vegetable protein is readily absorbed by the body [3,4,5]. In the natural environment, plants as sessile organisms are subject to various pests, diseases, and abiotic stresses, which have a large negative impact on their growth [6]. The best-known abiotic stresses are drought, heat, cold, and flooding. However, the effects of high relative humidity (RH) on crops have not been well studied, although high RH is not limited to tropical climate zones [7]. Given the high nutritional and commercial value of quinoa, it is important to study the adverse effects of RH stress on the quality and growth patterns of quinoa seedlings. Most studies on stress effects in quinoa focused on more-common abiotic stresses, such as drought and flooding, and the effects of high RH should be given more attention to fill the current research gaps.

High RH (RH ≥ 85%) is common in controlled environments and is not uncommon in nature. Plants grown at high RH and low RH (≤60%) often exhibit rapid wilting characteristics [8,9,10]. In addition, high RH affects plant growth [11,12], transpiration and nutrient uptake [13,14], and leaf temperature [15,16]. Numerous studies have shown that the flower number, leaf number, biomass, and flowering time of plants are not significantly affected by moderate-to-high RH levels [17,18,19,20,21]. However, stem length and leaf area tend to increase at high RH [22,23,24,25]. Although leaf biomass is generally unaffected by RH, the leaves of plants grown at high RH tend to be thinner and have reduced tissue density, resulting in increased specific leaf area [26,27,28]. High and low RH levels reduce the density of leaf veins in plants [29], which affects the water transport from the xylem to the stomatal cavities, leading to a reduction in the water transport capacity [30]. Leaf duct size and water-use efficiency also decrease [27]. Most crops grown in high RH environments have a higher number of stomata [31,32,33,34,35]. Moreover, leaves developed under high RH conditions have poorer stomatal responses and lower stomatal closure levels (consequently, abnormal stomata function) than those developed in moderate RH conditions, which have normal stomata function [36]. Impaired stomatal function under high RH further leads to greater susceptibility to biotic stress, as microorganisms can more easily enter the leaf tissues [37]. High RH also affects the levels of various plant hormones. For example, abscisic acid (ABA) levels are reduced under high RH conditions [38,39,40], whereas ethylene, cytokinin, and gibberellin levels are increased in leaf tissues under high RH conditions [36,40]. Therefore, it is particularly important to study the morphological, physiological, and molecular responses of quinoa to high RH stress.

Transcriptomics is an important research method used to characterize the entire set of transcripts and, thus, obtain information on gene expression in an organism. The metabolome is a direct reflection of an organism’s phenotype. Combining both approaches in order to analyze associations among genes and metabolites is currently an important and popular research method for studying plant stress resistance [41,42,43]. Although studies of plants under high RH stress have been reported, they mostly focused on horticultural plants and only examined the effects on their morphology and physiology. This study aimed to investigate the physiological response of quinoa to high RH and identify the respective molecular mechanisms involved. We combined metabolomics and transcriptomics with statistical measurements of morphological and physiological indicators to understand the responses of quinoa seedlings under high RH stress and their regulation at the transcriptional level and to provide a theoretical basis for quinoa stress resistance research.

## 2. Materials and Methods

### 2.1. Study Materials and High RH Treatment

Twenty independently selected lines of quinoa from Yunnan Agricultural University were used as primary screening materials and planted at the Modern Agricultural Education and Research Base of Yunnan Agricultural University in Xundian County, Kunming, China (102°41′ E, 25°20′ N). Uniform, full, intact seeds were selected from each of the 20 varieties, sterilized with 0.2% HgCl_2_ (All-Style Gold Biotechnology Co., Beijing, China), and placed in a light incubator at a constant temperature (25 °C) until they appeared dew white. The seeds were seeded evenly in 50-hole seedling trays (54 × 28 × 12 cm) in a greenhouse (16 h light/8 h dark). The sowing depth was 2–3 cm. We used two trays for each material control and two trays for each treatment, ensuring that there were approximately 250 seedlings in each tray. The seedlings were grown under an average temperature (day/night, 25 ± 1/18 ± 1 °C), normal RH (50%) and photosynthetically active radiation (2000 Lx) in loamy soil:humus:organic fertilizer = 1:1:1, using conventional cultivation management techniques. When the cotyledons were fully opened, we selected uniformly growing seedlings as the experimental material. The seedlings were transported to controlled greenhouses and exposed to normal RH (50 ± 2%, control group) or high RH (90 ± 2%, treatment group); the remaining experimental conditions were the same as in the previous planting. On Day 5 of treatment, we selected Dianli-969, which showed leaf wilting and slight discoloration, as the sensitive variety, and Dianli-439, which grew vigorously without wilting, as the resistant variety. On Day 14 of treatment, when irreversible decay began to occur in the sensitive variety (Dianli-969), the seedling leaves of the treatment and control groups of Dianli-969 and Dianli-439 were sampled separately, snap-frozen in liquid nitrogen, and stored at −80 °C. Three biological replicates and three technical replicates were included in this study. More details are provided in Table 1.

### 2.2. Analysis of Morphological and Physiological Indicators

Seedlings of the two varieties in the control and treatment groups were sampled separately (three replicates). The height, leaf area, and dry and fresh weights of the above-ground parts were determined. Height (i.e., the distance from the base to the top of the uppermost spreading leaf) was measured using Vernier calipers. Leaf area was measured using a TPYX-A instrument (Zhejiang, China, https://www.tpyn.net, accessed on 10 May 2022). The seedlings were washed directly after collection to determine fresh weight using a digital balance (sensitivity: 0.0000 g). The seedlings were deactivated at 110 °C for 30 min and dried to a uniform weight at 80 °C. The dry weights of the roots and shoots were measured to calculate the root-to-crown ratio. The chlorophyll content was determined according to the method reported by Shah et al. [44]. The leaves were cut into small pieces; 0.25 g of the sample was weighed, and 5 mL of 80% acetone was added for extraction under dark conditions. When the leaves had turned completely white, the supernatant was collected by centrifugation (8000× *g*, 10 min) and the absorbance of chlorophyll A and B was recorded at 663.6 nm and 646.6 nm, respectively. The ABA and malondialdehyde (MDA) contents and catalase (CAT), peroxidase (POD), and superoxide dismutase (SOD) activities were measured using kits (Nanjing Jiancheng Bioengineering Institute, Nanjing, China) according to the manufacturer’s instructions.

### 2.3. Metabolite and Transcript Determination and Analysis

#### 2.3.1. Metabolite Extraction and Liquid Chromatography-Mass Spectrometry Analysis

Samples were extracted according to the method reported by Chen et al. [45]. Freeze-dried samples were crushed using zirconia beads in a mixer mill (MM 400, Retsch, Haan, Germany, https://www.retsch.cn/) at 30 Hz for 1.5 min. The powder (100 mg) was weighed and extracted overnight at 4 °C with 1.2 mL of 70% aqueous methanol. Centrifugation at 12,000× *g* for 10 min and filtration through a SCAA-104 membrane (0.22 μm pore size; ANPEL, Shanghai, China) were carried out for subsequent LC-MS analysis. The analytical conditions used were set as described by Chen et al. [45]. All metabolites were screened using ultra-performance liquid chromatography (UPLC)-tandem mass spectrometry (MS/MS). UPLC analysis was conducted with the Shim-pack UFLC Shimadzu CBM30A instrument (Shimadzu, Kyoto, Japan) equipped with an Acquity UPLC HSS T3 C18 column (1.8 μm, 2.1 × 100 mm; Waters, Milford, MA, USA). The column temperature was 40 °C, and the mobile phase consisted of water and acetonitrile, both containing 0.04% acetic acid. Gradient elution was performed at a flow rate of 0.40 mL·min^−1^; the injection volume was 5 μL. MS/MS analysis was performed using an Applied Biosystems 4500 QTRAP system (Thermo Fisher Scientific, Waltham, MA, USA).

#### 2.3.2. Qualitative and Quantitative Analysis of Metabolites

We used the self-built Metware database and a public database (http://metware.com, 10 June 2022). Metabolites were quantified using the multiple reactions monitoring mode in a triple quadrupole mass spectrometer (AB Scientific, Waltham, MA, USA). Orthogonal partial least-squares discriminant and multivariate statistical analyses with supervised pattern recognition were used for identification of the metabolites. Hierarchical clustering and heatmaps of the expressed metabolites were generated using the “pheatmap” R package. Variable importance in projection (VIP) ≥ 1 and |log2 (fold change)| ≥ 2 were selected as thresholds to identify differentially accumulated metabolites (DAMs) for subsequent analysis.

### 2.4. Transcript Profiling and Data Analysis

#### 2.4.1. RNA Extraction and Sequencing 

Total RNA was extracted from grain samples using Trizol (Invitrogen, Thermo Fisher Scientific, https://www.thermofisher.cn/) according to the manufacturer’s instructions. RNA integrity and purity were determined using 1% agarose gels and a NanoPhotometer spectrophotometer (Implen, München, Germany), respectively. RNA concentration was calculated using the QubitRNA Assay kit and Qubit 2.0 fluorometer (Life Technologies, Thermo Fisher Scientific, https://www.thermofisher.cn/). RNA integrity was also confirmed using the RNA Nano 6000 Assay kit and 2100 Bioanalyzer system (Agilent Technologies, Santa Clara, CA, USA). Then, 3 μg RNA per sample was used as the input material for sequencing. Sequencing libraries were constructed using the NEBNext Ultra RNA Library Prep Kit from Illumina (New England Biolabs, Ipswich, MA, USA). Total RNA was extracted. Ribosomal RNA was removed. The mRNA was fragmented. The fragments were used as templates for cDNA synthesis, followed by purification of the double-stranded cDNA using AMPure XP beads and fragment size selection using AMPure XP beads (150–200 bp fragments). PCR amplification was then performed using Phusion High-Fidelity DNA polymerase, universal PCR primers, and the Index (X) Primer. The PCR products were purified using the AMPure XP system (Beckman Coulter, Indianapolis, IN, USA), and their quality was assessed in a 2100 Bioanalyzer (Agilent Technologies, https://www.agilent.com.cn/). Reads containing adapters or poly-N sequences, as well as low-quality reads were removed from the raw data to obtain clean reads in the FASTQ format. The Q20 and Q30 values and GC contents were determined according to conventional methods.

#### 2.4.2. Identification of Differentially Expressed Genes and Enrichment Analysis

For data analysis, the obtained paired reads were mapped to the wax gourd (B227) genome (v1, WG_genome.fa.gz at http://cucurbitgenomics.org/, accessed on 20 June 2022) using HISAT2 with default parameters [46]. The mapped reads across each gene were counted using the featureCounts algorithm [47], and the fragments per kilobase million (FPKM) were calculated. Principal component analysis (PCA) was performed to compare the FPKM values of the expressed genes within grains using the “prcomp” R function. Hierarchical clustering and heatmaps of the expressed genes were generated using the “pheatmap” R package. Differentially expressed genes (DEGs) were identified using the method reported Mortazavi et al. [48]. DEGs were identified using a false discovery rate threshold of <0.05. Gene ontology (GO) and Kyoto Encyclopedia of Gene and Genomes (KEGG) pathway enrichment analyses were then performed using the “GOseq” R package [49] and KOBAS software [50], respectively.

### 2.5. Joint Transcriptome and Metabolome Analysis

The bulk data were normalized and statistically analyzed to establish relationships at various biomolecular levels. The DEGs and DAMs were mapped to the Kyoto Encyclopedia of Genes and Genomes (KEGG) pathways to identify shared pathways and were correlated to explain the regulatory mechanisms between molecules at different levels. TBtools [51] was used to generate heatmaps of key enzymes and genes in the pathways.

### 2.6. Transcription Factor Identification, Weighted Gene Co-Expression Network Analysis, and Gene Network Visualization

Transcription factor families were identified and annotated using the iTAK software (IAITAM, Canton, OH, USA) [52,53]. The Multiple Expectation Maximization for motif Elicitation (MEME) (http://meme-suite.org/tools/meme, accessed on 15 October 2022) tool was used to predict conserved motifs in AP2/ERF family proteins [54]. After discarding undetectable and low-expression genes, a co-expression network of the DEGs was generated using the Weighted Gene Co-Expression Network Analysis (WGCNA) package in R. Co-expression modules were identified using automatic network construction functions (blockwiseModules) with default parameters. Soft beta thresholds were selected, and co-expression modules were delineated using the “pickSoftThreshold” R function. A topological overlap matrix, which reflects the similarity in terms of the commonality of the nodes they connect to between two nodes, was constructed by calculating a soft-threshold-based adjacency matrix. A gene clustering tree obtained after hierarchical clustering was cut using the dynamic shear tree algorithm, and colors were randomly assigned to all modules for visualization. Eigenvectors were calculated for each module using PCA and were used to calculate the correlations between modules and traits. Interaction relationships in the STRING protein interaction database (http://string-db.org, accessed on 20 October 2022) were used to analyze differential protein interactions in a protein–protein network (PPI). A network module map was generated using CytoScape (v. 3.9.1) [55], with all analytical parameters set to default.

### 2.7. Quantitative Reverse Transcription-PCR

RNA was extracted from samples from each group separately and used for reverse transcription (RT)-qPCR. Gene-specific primers were designed using Primer Premier 5 (Supplementary Materials Table S1) (https://macdownload.informer.com/primer-premier/, accessed on 5 November 2022). TUB-6 was included as an internal reference gene. qPCRs were run in triplicate using PerfectStart SYBR qPCR Supermix (TransGen Biotech, Beijing, China) in a StepOnePlus instrument (Applied Biosystems, Foster City, CA, USA). Relative target gene expression levels were determined using the 2^–ΔΔCT^ method [56]. The gene expression values represent the mean of three independent biological replicates.

### 2.8. Statistical Analysis

All data were processed and statistically analyzed using Excel 2010 (Microsoft Corporation, Redmond, Washington, WA, USA) and SPSS 26.0 for Windows (IBM Corp., Armonk, NY, USA). Significant differences were identified using a two-way ANOVA test. Results with *p* < 0.05 were deemed statistically significant. All data in the figures and tables are expressed as the mean ± the standard error (*n* ≥ 3).

## 3. Results

### 3.1. Changes in Morphological Indicators under High RH Stress

We compared the changes in morphological indicators between the two varieties after 5 and 14 days under high RH stress (Table 2). A phenotype map of the performance of quinoa under high RH stress is provided in Supplementary Materials Figure S1. After 5 days of high RH stress, seedlings showed wilting and growth retardation in the treatment groups of both varieties compared to the control groups, although these changes were not significant. There were no significant differences in plant height, leaf area, fresh weight, and root-to-shoot ratio between the treated samples and control groups. In contrast, the dry weight did not significantly differ between the varieties, but it did significantly differ between the treatments. After 14 days of high RH stress, seedlings of both varieties showed no significant differences in plant height, leaf area, fresh weight, and dry weight, but plants of the sensitive variety were significantly shorter than control plants. The root–shoot ratio of both varieties did not change significantly after 5 days of stress, but after 14 days of stress, seedlings of the resistant variety in the treatment group were significantly higher than control seedlings, whereas the opposite was true for the sensitive variety. These findings indicated that the sensitive-type seedlings were wilted, their growth was stunted, and the dry matter accumulation was affected by Day 5 of HR stress. Moreover, after 14 days of high RH stress, wilting and growth retardation were more pronounced in the sensitive variety, whereas seedlings of the resistant variety were less damaged.

### 3.2. Changes in Physiological Indicators under High RH Stress

We examined changes in the physiological parameters of both varieties after 5 and 14 days under high RH stress (Table 3). The chlorophyll content was significantly decreased in the treated groups compared to the control groups after 5 days of high RH stress. However, the chlorophyll content in the treatment group of the highly resistant variety was higher than that in the sensitive variety. The MDA content in the sensitive variety treatment group was significantly higher than that in the resistant variety treatment group, and both were higher than those in the control groups. The SOD and CAT activities were higher in the resistant variety than in the sensitive variety, and both were higher than those in the control group, whereas the POD activity was higher in the sensitive variety than in the resistant variety, and both were higher than the levels in the control groups.

After 14 days of high RH stress, chlorophyll continued to decrease and was significantly lower in the treatment than in the control groups, but it was significantly lower in the sensitive than in the resistant variety. Both the MDA content and POD activity were higher in the sensitive variety than in the resistant variety and were significantly higher in the treatment than in the control groups. In contrast, the SOD and CAT activities were higher in the resistant than in the sensitive variety, and both were higher in the treatment than in the control groups. Noteworthily, ABA, which was not significantly different between the resistant and sensitive varieties after 5 days of high RH, was significantly higher in the resistant than in the sensitive variety at 14 days of treatment. Therefore, we concluded that, in addition to changes in the wilting and stunting of the seedlings by Day 5 of high RH stress, there were also significant changes in the intrinsic physiology of these seedlings, with notable changes in the chlorophyll and MDA contents. In contrast, the resistant variety showed significantly smaller changes in ABA content and higher enzymatic activity after 14 days of treatment, thereby demonstrating greater tolerance and outperforming the sensitive variety in all indicators.

### 3.3. Metabolic Profiling of Quinoa Seedlings under High RH Stress

**Figure 1:**
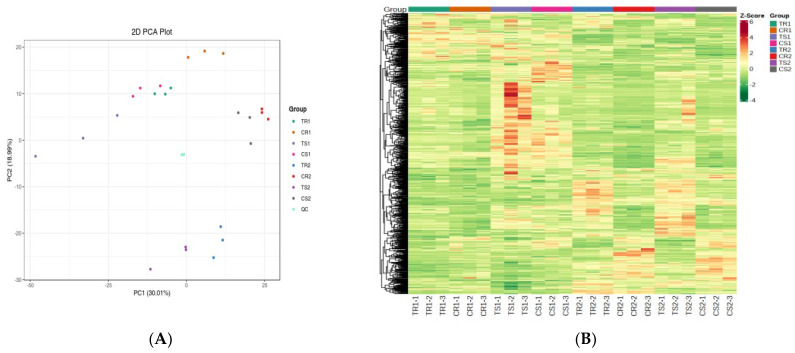
Overview of the metabolome changes in quinoa seedlings under high RH stress. (**A**) PCA plot of the samples. (**B**) Hierarchical clustering heatmap of the DAMs.

To gain insight into the metabolic changes involved in high RH stress, we performed metabolomics analysis of quinoa seedlings after high RH treatment (5 and 14 days) using an ultra-high-performance liquid chromatography-tandem mass spectrometry platform. In total, 10,60 metabolites were detected in 24 samples, including amino acids and their derivatives (119), phenolic acids (157), nucleotides and their derivatives (62), flavonoids (167), quinones (14), lignins and coumarins (29), alkaloids (112), terpenoids (48), organic acids (84), lipids (173), and others (95). We performed PCA on the data to obtain a preview of the entire metabolome (Figure 1A). The first and second first principal components explained 30.01% and 18.99% of the metabolite variation in all samples, respectively. Moreover, eight groups of samples were well differentiated, but the differences among them were not statistically significant; thus, the changes in the metabolites of quinoa seedlings varied considerably under different RH conditions and could be used for further analysis. Based on the relative metabolite concentrations, hierarchical clustering analysis of the samples showed that the metabolite content was significantly higher in the sensitive variety than in the highly resistant variety after 5 days of treatment (Figure 1B). In addition, the metabolite content was significantly increased in the treatment groups compared with the control groups.

### 3.4. Significant DAMs and Enrichment Analysis 

To gain insight into the differences in the metabolites between the subgroups, |log2(fold change)| ≥ 2 and VIP ≥ 1 were used to identify significant DAMs. We divided the 24 samples into 12 comparison groups: treatment (T) vs. control (C), highly resistant (R) vs. sensitive (S) variety, and 5 days (1) vs. 14 days (2), as detailed in Supplementary Materials Table S2. DAM expression was upregulated in the treatment groups compared to the control groups at both time points and in both varieties. There were 210 DAMs in TS1 vs. CS1 (172 up, 38 down), 315 in TS2 vs. CS2 (216 up, 99 down), 156 in TR1 vs. CR1 (123 up, 33 down), and 298 in TR2 vs. CR2 (224 up, 74 down). DAM expression was upregulated in the sensitive variety compared to the resistant variety at both 5 days and 14 days. There were 241 DAMs in TR1 vs. TS1 (201 up, 40 down) and 159 in TR2 vs. TS2 159 (105 up, 54 down). DAMs were significantly downregulated at 14 days in both varieties, except in the treatment group of the resistant variety.

**Figure 2:**
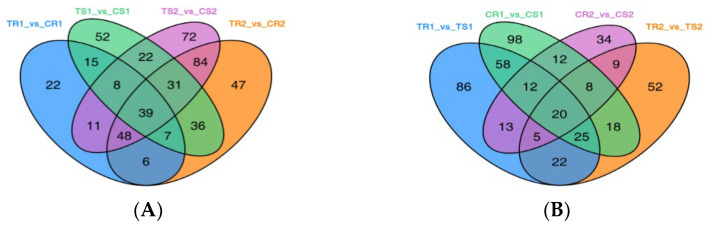

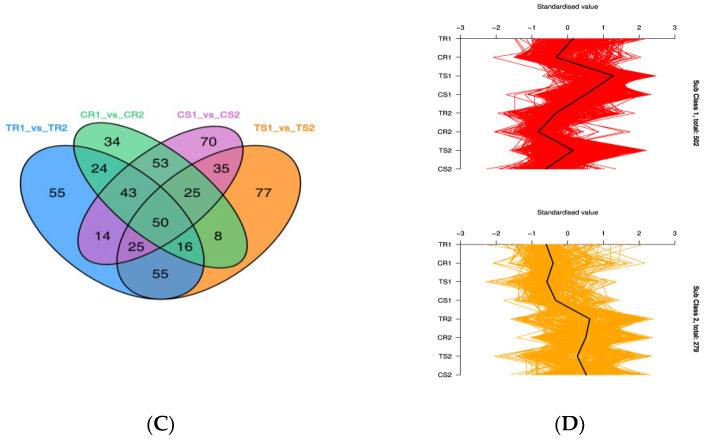
Venn diagram and K-means plot of the DAMs. (**A**–**C**) Venn diagrams of the DAMs for each comparison group. (**D**) K-means plot of the DAMs.

According to Venn diagram analysis, 100 DAMs were shared between the treatment and control groups of the highly resistant variety upon 5 and 14 says of treatment (Figure 2A). Similarly, 100 DAMs were shared between the treatment and control groups of the sensitive variety on Days 5 and 14 of treatment (Figure 2A). The highly resistant and sensitive varieties shared 72 DAMs after Days 5 and 14 of treatment and 52 when untreated (Figure 2B). Moreover, 133 and 135 DAMs were shared between Days 5 and 14 of treatment in the resistant and sensitive varieties, respectively (Figure 2C).

K-means clustering was used to examine trends in the relative DAM contents in the different subgroups (Figure 2D). Dividing the DAMs into two clusters, we found that the DAMs in Cluster 1 were significantly decreased on Day 14, whereas the opposite was true in Cluster 2. In Cluster 1, the sensitive variety was the most enriched in DAMs at Day 5, and in Cluster 2, the resistant variety was the most enriched in DAMs at Day 14. Notably, in Cluster 1, the most-abundant DAMs were lipids (109) and flavonoids (88). In Cluster 2, the most-abundant DAMs were phenolic acids (56) and flavonoids (44).

The top 20 DAMs between groups are listed in Supplementary Materials Table S3. The highly resistant variety at 5 and 14 days after treatment with high RH showed a significant accumulation of terpenoids, organic acids, flavonoids, nucleotides and derivatives, amino acids and derivatives, lipids, and phenolic acids compared to the control, whereas the sensitive variety showed significant enrichment of phenolic acids, lipids, alkaloids, and flavonoids. Phenolic acids, lignans, coumarins, alkaloids, nucleotides and derivatives, quinones, and flavonoids were significantly accumulated in the highly resistant variety at 5 and 14 days after high RH treatment compared to the sensitive variety. After 5 and 14 days of high RH treatment, the DAMs in the highly resistant variety were mainly lipids, phenolic acids, and flavonoids compared to the control, and those in the sensitive variety were mainly terpenoids and phenolic acids. The same results are shown in Supplementary Materials Figure S2.

As shown in Supplementary Materials Figure S3 and Table S4, the two varieties had different significantly enriched pathways in response to high RH stress. The main differences between the two varieties were in phenylalanine metabolism, the biosynthesis of various alkaloids, caffeine metabolism, ubiquinone and other terpenoid-quinone biosynthesis, and flavone and flavanol biosynthesis. Notably, the resistant variety was significantly enriched in DAMs related to arginine biosynthesis at both 5 and 14 days after high RH treatment, whereas the sensitive variety was not. On Day 14, flavone and flavanol biosynthesis were significantly enriched in both varieties. These results indicated that the DAMs differed between the varieties, treatments, and stress periods. These metabolites were significantly upregulated in the treatment groups of both varieties in response to high RH stress, but the changes were greater in the sensitive than in the resistant variety. Lipids and flavonoids were detected in large quantities in the sensitive variety after 5 days of high RH stress. In contrast, phenolic acids and flavonoids were detected in larger quantities in the highly resistant variety after 14 days of high RH stress. These findings indicated that the highly resistant and sensitive varieties accumulated different DAMs and activated different metabolic pathways in response to high RH stress.

### 3.5. Transcriptional Profiling of Quinoa Seedlings under High RH Stress

Twenty-four samples were sequenced, yielding an average of approximately 44.5 million high-quality clean reads per library. An overview of the RNA-seq data is shown in Supplementary Materials Table S5. The percentage of clean reads mapped to the quinoa reference genome ranged from 88.60% to 95.87%. The average Q20 and Q30 values were 97.76% and 93.49%, respectively. In total, 59,086 genes were annotated in the NCBI NR database, 37,101 genes in Swiss-Prot, 41, 125 genes in Gene Ontology (GO), 52,602 genes in Eukaryotic Orthologous Groups, 47,860 genes in PFAM, 57,905 genes in TrEMBL, and 42,891 genes in KEGG (Supplementary Materials Table S6). The KEGG functionally annotated genes were mainly involved in 142 pathways (Supplementary Materials Table S7), and GO functions were annotated in terms of molecular function (MF), biological process (BP), and cellular component (CC).

### 3.6. Identification of DEGs and GO Term and KEGG Pathway Enrichment Analysis

DEGs between the subgroups were identified using |log2(fold change)| ≥ 1 and FDR < 0.05 as the criteria. In total, we identified 13,095 DEGs. DEG statistics for each comparison group are provided in Supplementary Materials Table S8. There were 5683 DEGs in TR1 vs. TS1 (2495 down, 3188 up), 821 in TR2 vs. TS2 (392 down, 429 up), 1469 in TR1 vs. CR1 (1003 down, 466 up), 6227 in TR2 vs. CR2 (3230 down, 2997 up), 6038 in TS1 vs. CS1 (3400 down, 2638 up), 5527 in TS2 vs. CS2 (3232 down, 2295 up), 3429 in TR1 vs. TR2 (1735 down, 1694 up), and 3129 in TS1 vs. TS2 (2081 down, 1048 up). After high RH treatment, DEGs were predominantly upregulated in both varieties, and DEGs were significantly more strongly upregulated in the resistant variety than in the sensitive variety.

To functionally characterize the DEGs, GO enrichment analysis of each group was conducted. The DEGs were mainly involved in the cellular anatomical entity (CC), cellular process (MF), catalytic activity (BP), binding (MF), and metabolic process (BP) (Supplementary Materials Figure S4). As shown in Supplementary Materials Figure S5, among the top 50 GO terms of DEGs after high RH stress, the resistant variety was mainly enriched in terms related to photosynthesis and response to organonitrogen compounds, plant-type cell wall, and enzyme inhibitor activity in the BP, CC, and MF categories, respectively. The sensitive variety was mainly enriched in terms related to photosynthesis, plant-type cell wall, and enzyme inhibitor activity in the BP, CC, and MF categories, respectively. In contrast, the top 50 GO terms of DEGs between the resistant and sensitive varieties were mainly related to the mitotic cell cycle, microtubule cytoskeleton, tubulin binding, benzene-containing compound metabolic process, and magnesium ion binding in the BP, CC, and MF categories.

Supplementary Materials Table S9 and Figure S6 show the metabolic pathways that were significantly altered in each group. KEGG analysis showed that, after high RH treatment, genes related to the biosynthesis of secondary metabolites; flavonoid biosynthesis; MAPK signaling pathway-plant; glucosinolate biosynthesis; nitrogen metabolism; alpha-linolenic acid metabolism; glyoxylate and decarboxylate metabolism; plant hormone signal transduction, cutin, suberin, and wax biosynthesis; plant–pathogen interaction; benzoxazinoid biosynthesis; zeatin biosynthesis; and ABC transporters were significantly enriched in both varieties. Both varieties were significantly enriched in genes related to photosynthesis-antenna proteins; metabolic pathways; and the biosynthesis of secondary metabolites pathways. Circadian rhythm-plant; isoflavonoid biosynthesis; glycerophospholipid metabolism; cutin, suberin, and wax biosynthesis; ABC transporters; glucosinolate biosynthesis; alpha-linolenic acid metabolism; zeatin biosynthesis; and decarboxylate metabolism were significantly enriched after 5 and 14 days of treatment.

**Figure 3:**
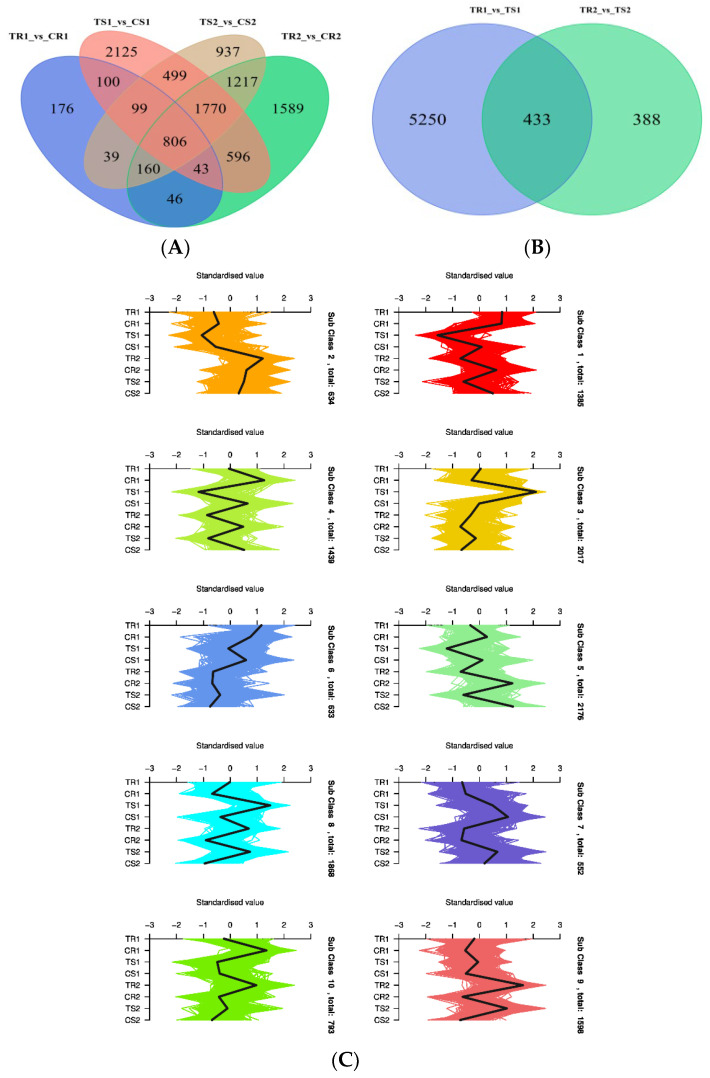
Venn diagrams (**A**,**B**) and K-means plot (**C**) of the DEGs.

As can be seen in Figure 3, in the highly resistant variety, there were 1055 (Figure 3A) DEGs between the treatment and control groups at both time points. In the sensitive variety, there were 3174 DEGs between the treatment and control groups at both time points (Figure 3B). There were 433 DEGs between resistant and sensitive varieties at both time points (Figure 3C). K-means clustering of the DEGs revealed 10 clusters: there were 1385 DEGs in Cluster 1, 634 in Cluster 2, 2017 in Cluster 3, 1439 in Cluster 4, 2176 in Cluster 5, 633 in Cluster 6, 552 in Cluster 7, 1868 in Cluster 8, 1598 in Cluster 9, and 793 in Cluster 10. The genes in Clusters 1, 2, 6, 8, and 10 were significantly differentially expressed in the resistant variety under high RH conditions, which may serve as potential resistance marker genes in quinoa under high RH stress conditions.

### 3.7. Changes in Gene Expression and Metabolite Accumulation in Quinoa Seedlings under High RH Stress

**Figure 4:**
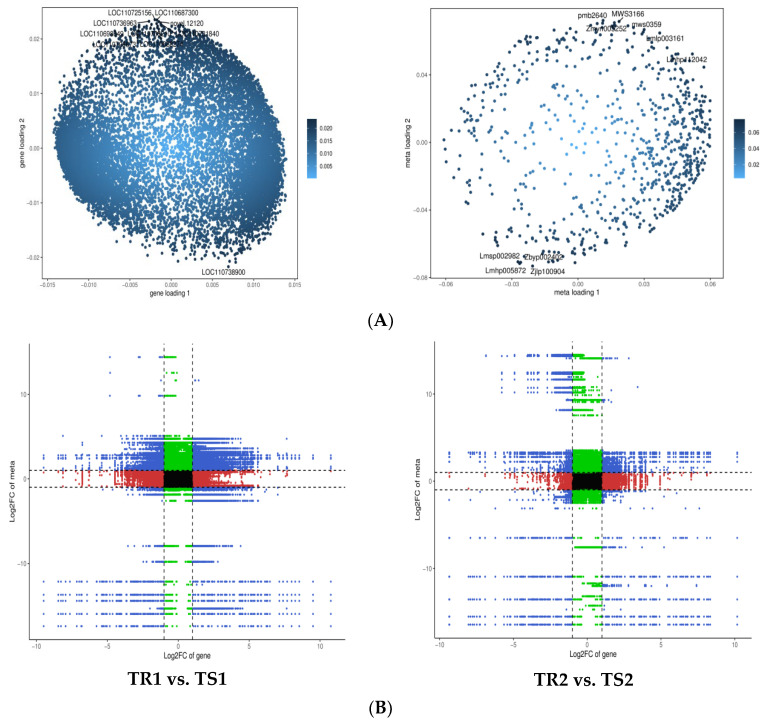

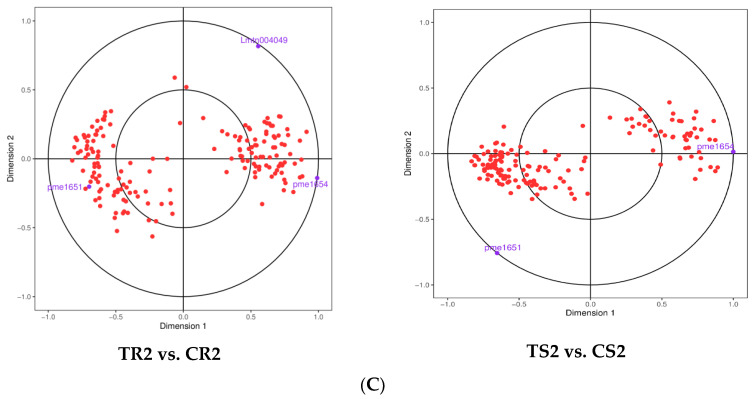
Schematic diagram of the combined analysis of DEGs and DAMs. (**A**) Left, transcriptome loading plot; right, metabolome loading plot. (**B)** Correlation analysis nine-quadrant plot. (**C**) Canonical correlation analysis.

We performed a combined transcriptome and metabolome analysis to further understand the mechanism underlying the response of quinoa to high RH stress. All the differential genes and metabolites were selected to establish O2PLS models, and the variables with higher correlation and weight in different data groups were initially judged by the loading diagram. Important variables affecting other histological features were screened out. The results of O2PLS modelling are shown in Figure 4A. Changes in the transcriptome strongly affected the metabolome. Supplementary Materials Table S10 lists the top 10 genes that strongly affected the metabolome and the top 10 metabolites that significantly affected the transcriptome. Notably, 3 of the 10 genes affecting the metabolome (LOC110725156, LOC110706673, and LOC110731840) were involved in the plant–pathogen interaction pathway (ko04626), and 4 and 2 of the 10 metabolites affecting the transcriptome were lipids and alkaloids, respectively (see Supplementary Materials Table S11 for details).

Expression correlation analysis revealed strong positive and negative correlations (Figure 4B) between numerous DEGs and DAMs (R > 0.8) in the comparison of TR1 vs. TS1 and TR2 vs. TS2, suggesting that more-numerous DAM changes may be positively and negatively regulated by genes in the resistant variety. Figure 4C shows the results of the canonical correlation analysis of the DAMs and DEGs in the phytohormone signaling pathway for both varieties after 15 days of HR stress. Overall, pem1651 (indole-3-acetic acid (IAA)) and pem1654 (jasmonic acid (JA)) were found to be highly correlated with most genes after high RH treatment, and pem1651 was significantly more correlated with DEGs in the resistant than in the sensitive variety. Metabolite lmtn004049 (ABA) differed significantly after treatment compared with the control in the resistant variety, whereas such a difference was not observed in the sensitive variety.

### 3.8. Response of Plant Hormone Signal Transduction Pathways to High RH Stress

**Figure 5:**
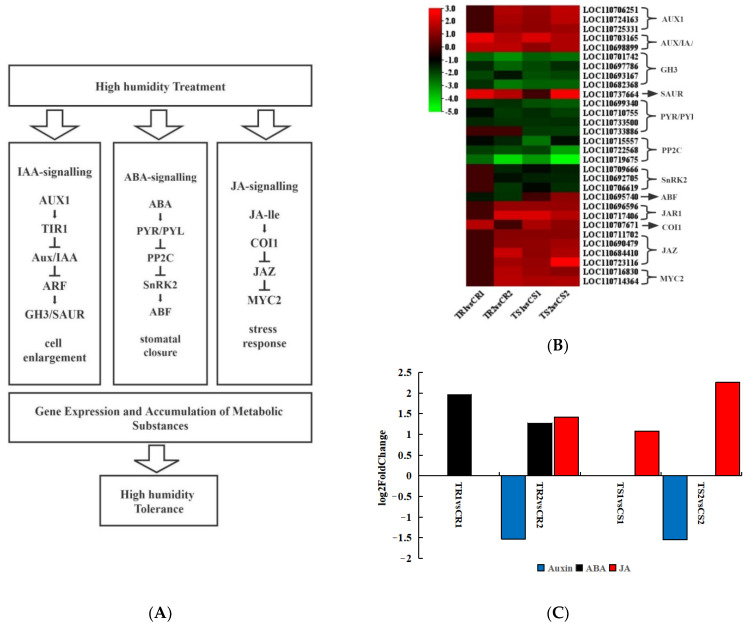
Changes in gene expression levels and metabolite contents in the hormone signaling pathway. (**A**) The pathway in auxin, ABA, and JA signaling networks. (**B**) Heatmap of the relative expression levels of genes normally involved in this pathway. (**C**) Metabolite abundance in the auxin, ABA, and JA signaling pathways in response to high RH stress.

A comprehensive analysis of the DEGs and DAMs under high RH treatment revealed differential changes in pathways such as the thioglucoside biosynthesis pathway (ko00966), glycerophospholipid metabolism pathway (ko00564), metabolic pathway (ko01100), starch and sucrose metabolism pathway (ko00500), diterpene biosynthesis pathway (ko00904), and phytohormone signaling pathway (ko04075). In particular, the phytohormone signaling pathway was significantly altered in both the resistant and sensitive varieties after 14 days of high RH stress (Figure 5A). As shown in Figure 5C, auxin was not significantly altered on Day 5, but was significantly decreased in both varieties after 14 days of high RH stress. The resistant variety showed a significant increase in ABA at both Days 5 and 14, whereas the sensitive variety showed no significant change at both Days 5 and 14 days. In the resistant variety, JA increased significantly only on Day 14, whereas in the sensitive variety, it was significantly increased at both Days 5 and 14.

Figure 5B shows the expression heatmap of genes related to three important plant hormones (ABA, JA, and IAA) in response to high RH stress. The auxin carrier protein AUX1 (LOC110706251, LOC110724163, and LOC110725331) was significantly downregulated in the treatment groups, as were the auxin/IAA response proteins (LOC110703165 and LOC110698899) and small auxin-upregulated RNA family of proteins (LOC110737664). However, the auxin-responsive GH3 protein family (LOC110701742, LOC110697786, LOC110693167, and LOC110682368) was significantly upregulated. ABA-related genes, such as the abscisic acid receptor family PYR/PYL (LOC110699340, LOC110710755, and LOC110733500) and protein phosphatase 2C (LOC110715557, LOC110722568, and LOC110719675), were mostly upregulated. Serine/threonine-protein kinases (LOC110709666, LOC110692705, and LOC110706619) and the ABA-responsive element-binding factor (LOC110695740) were significantly upregulated under high RH stress. Similar to the auxin-related genes, JA-related genes were all downregulated, except in the resistant variety at Day 5. For example, JA-amino synthetase (LOC110696596 and LOC110717406), coronatine-insensitive protein 1 (LOC110707671), jasmonate ZIM domain-containing protein (LOC110711702, LOC110690479, LOC110684410, and LOC110723116), and transcription factor MYC2 (LOC110716830 and LOC110714364) were downregulated. Based on the combined results of the transcript and metabolite analyses, we suggest that quinoa seedlings respond to high HR stress primarily by regulating hormone signaling, with the most-pronounced changes occurring in the auxin-, ABA-, and JA-related pathways, which regulate gene expression and metabolite accumulation.

### 3.9. Differentially Expressed Transcription Factors under High RH Stress

We next investigated the expression of the transcription factors of high RH stress. In total, 2825 DEGs encoding transcription factors were identified (Table 4). These differentially expressed transcription factors belonged to different families, such as AP2/ERF, bHLH, C2C2, C2H2, C3H, MYB, NAC, WRKY, and B3. Notably, five of the top 10 most-highly expressed transcription factors belonged to the AP2/ERF transcription factor family and were significantly upregulated in the treatment groups compared to the control groups, and nearly all AP2/ERF genes were identified as DEGs. We identified six motifs in these five AP2/ERF genes, as shown in Supplementary Materials Figure S7. LOC110689144 and LOC110734990 contained all six motifs, whereas LOC110730331 and LOC110706505 only had two. Notably, all genes harbored the AP2 domain. Since FAR1, bHLH, and AP2/ERF are known to be primarily associated with developmental and stress responses in plants, we suggest that the upregulation of transcription factors, particularly those related to phytohormone signaling, is an important mechanism underlying the response to high RH stress in quinoa seedlings.

### 3.10. Combined WGCNA and PPI Analysis to Screen Long-Acting Response Hub Genes in the Highly Resistant Variety

**Figure 6:**
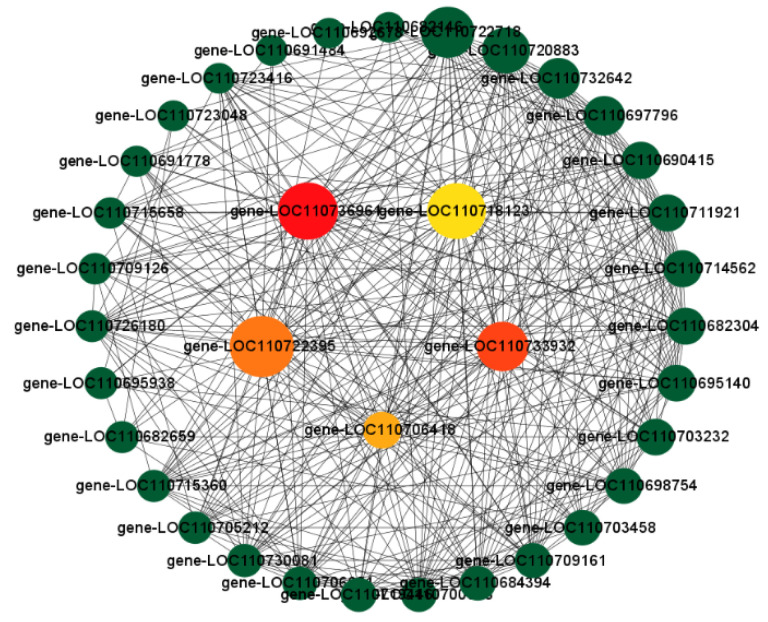
Candidate hub genes screened in the yellow module.

The genes remaining after filtering out the lowly expressed and stable genes were used to construct a WGCNA. As shown in Supplementary Materials Figure S8A, a total of 16 modules, which are given different colors, were detected. The genes in the modules were all highly relevant to the different samples. The yellow module was highly correlated with the resistant variety, with a correlation coefficient R of 0.92 and a *p*-value of 2e^−10^ (Supplementary Materials Figure S8B). Based on the kWithin values (representing gene connectivity within a module), 50 candidate hub genes were selected (Supplementary Materials Table S12). Moreover, the cytoNCA plugin in the CytoScape 3.9.1 software (https://cytoscape.org/, accessed on 20 October 2022) was used to identify the PPI network. Then, the hub genes were screened, as shown in Figure 6. The potential hub genes shown in Figure 6 include LOC110722395 (phosphoglycerate kinase, chloroplastic), LOC110733932 (chlorophyll synthase, chloroplastic-like), LOC110706418 (serine hydroxy methyltransferase, mitochondrial), LOC110718123 (heat shock factor protein HSF24-like), LOC110736961 (Euphorbiaceae, mitochondrial), and LOC110736961 (E3 ubiquitin-protein ligase ATL31-like).

### 3.11. Screening for Morphologically and Physiologically Important Candidate Genes Using WGCNA

**Figure 7:**
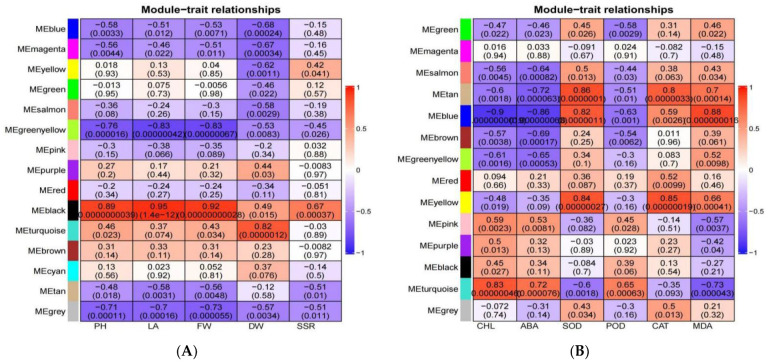
WGCNA plot of all genes. (**A**) Heatmap of association between gene co-expression network modules and agronomic traits; (**B**) Heatmap of association between gene co-expression network modules and physiological indicators.

After filtering all genes for undetectable or relatively low-expression genes (FPKM ≤ 1), WGCNA was performed to investigate the gene co-expression network. We correlated agronomic traits with genes to further understand the relationship between transcript accumulation and morphology for each module (Figure 7A). We found that the black module was highly correlated with plant height, leaf area, and fresh weight with correlation coefficients of 0.89, 0.95, and 0.92, respectively, and was significantly correlated (*p* < 0.001). To identify significant candidates for this module, we screened 27 genes with a gene significance (GS) ≥ 0.8 and module membership (MM) ≥ 0.8 (Supplementary Materials Table S13). Similarly, we correlated the physiological indicators as trait data with the modules (Figure 7B). We found that the blue module was highly correlated with chlorophyll, abscisic acid, malondialdehyde, and superoxide dismutase, with significant correlations (*p* < 0.001). We screened the blue module for 23 significant genes (absolute values of both MM and GS > 0.9) (Supplementary Materials Table S14). As shown in Supplementary Materials Tables S13 and S14, the candidate genes for the black module are mainly enriched in the protein processing in the endoplasmic reticulum, MAPK signaling pathway-plant, starch and sucrose metabolism, metabolic pathways, and biosynthesis of secondary metabolites pathways. Meanwhile, the candidate genes of the blue module are mainly enriched in the MAPK signaling pathway-plant, plant hormone signal transduction, glycerophospholipid metabolism, metabolic pathways, and biosynthesis of secondary metabolites pathways. It is not difficult to find that the MAPK signaling pathway-plant is enriched in both the black module (morphology) and the blue module (physiology), suggesting that its effect on morphology and physiology may be significant under high stress.

As shown in Table 5, among the candidate genes highly associated with morphology are stress proteins, the heat shock protein family, and calcium-related proteins. The most notable of the highly physiologically relevant candidate genes are the genes related to the basic components of the cell wall and the abscisic acid receptor genes.

### 3.12. Validation of the Transcriptome Data by RT-qPCR Analysis

**Figure 8:**
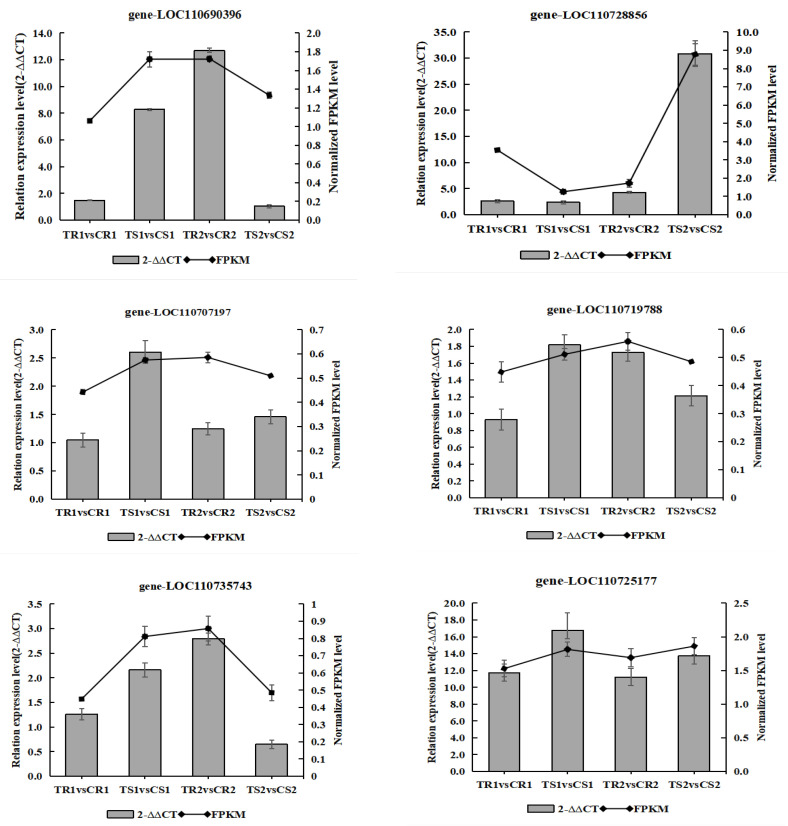

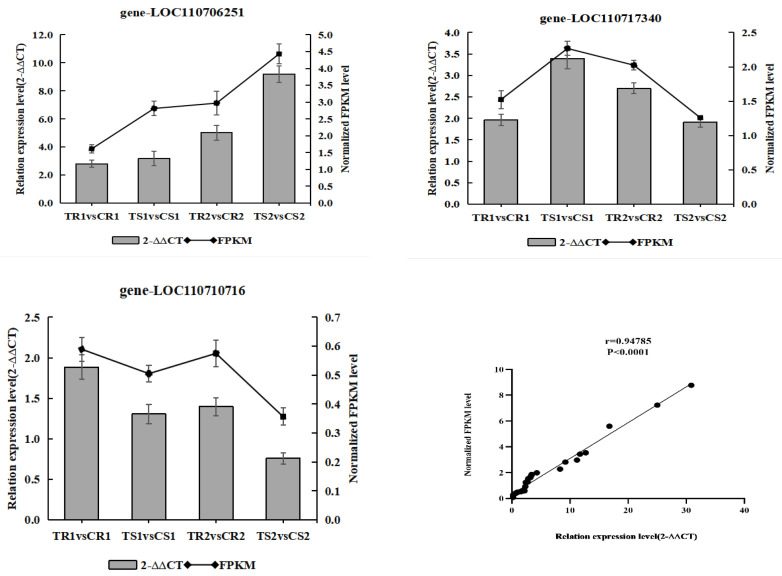
Verification of the RNA-seq results by RT-qPCR. Bars represent the means ± SEs (*n* = 3).

We selected nine genes in important pathways for RT-qPCR analysis, namely the ABA-related genes LOC110710716 and LOC110706251, JA-related gene LOC110725177, chlorophyll-related gene LOC110690396, CAT-related gene LOC110719788, cell wall development-related gene LOC110735743, ABA precursor and photoprotection-related gene LOC110717340, MADS transcription factor family and dormancy-related protein-coding gene LOC110728856, and the gene coding defense proteins in the cell wall LOC110707197. This genetic analysis validated the accuracy and reproducibility of the transcriptome data obtained. Overall, the results indicated that the RT-qPCR expression profile was consistent with that of the RNA-seq data (Figure 8), with a high correlation coefficient (R > 0.9), corroborating the reliability of the transcriptome data. Notably, the ABA-related gene LOC110706251 and JA-related gene LOC110725177 were expressed at higher levels in the sensitive than in the highly resistant material during both stress periods.

## 4. Discussion

Plant morphology (i.e., plant height, stem diameter, and leaf size) is generally significantly affected by high RH [23]. Prolonged exposure to 90% RH was reported to significantly increase stomatal conductance [57]; thus, stomata are directly influenced by ambient humidity [6], which in turn affects the leaf gas exchange response [58]. A previous study showed that an increase in RH results in larger leaf area and thinner leaves in roses, with stomata being more responsive in roses grown at 40% RH than in those grown at 60% RH [21]. Fanourakis et al. also found that plants grown at higher ambient humidity (95%) undergo increased water loss, which may be related to higher epidermal transpiration [32]. Nonetheless, some of these indicators are controversial. Watermelons were shown to be able to maintain leaf water content when under low-temperature stress and high RH by balancing water uptake and loss [59]. 

In this study, we found that high RH has a significant effect on the morphology of quinoa seedlings. Previous investigations have suggested that biomass is not significantly affected by high RH stress [20] and that stem length and leaf area increase [21,22]. This is not in line with our results, which revealed lower plant height, leaf area, dry weight, and fresh weight in quinoa seedlings under high RH stress for 14 days. In contrast, in a highly resistant variety, these changes were not significant at the beginning (Day 5) of the end (Day 14) of the stress period. These results suggest that the highly resistant variety is morphologically sturdier concerning high RH stress. Hence, we suggest that high RH lowers the evaporative demand, thus reducing the transpiration rate, despite the stomata being open [34,60]. These indicators will decline to lower levels than in control plants due to the reduced transpiration rate and xylem water flux, which further limits nutrient uptake by the root system [61], consequently limiting plant growth. Furthermore, reduced fresh weight may be related to the rate of water loss from the leaf epidermis, in which the reduced sensitivity of the stomata to closing stimuli and poorly developed epidermal waxes (i.e., higher cuticle permeability) can lead to water loss [62]. Our results further showed that the root-to-crown ratio increased under high RH, especially in the highly resistant variety, with significant increases over the control at both Days 5 and 14. We suggest that this event may result from increased root growth in the resistant variety under high RH stress. Indeed, previous studies showed that exposure to high RH can accelerate lateral root formation in rice [63], increase the number of root tips in rose birch [64], and increase root length in soybeans [65]. This observation also indirectly explains why the root-to-crown ratio of the sensitive variety decreased significantly on Day 14, since its root system may not have been as active and the secondary root production may not have been as efficient as in those of the resistant variety.

The physiological changes in quinoa seedlings under high RH stress are equally interesting. We found a significant decrease in the chlorophyll content under high RH stress, which may be explained by reduced chlorophyll biosynthesis and accelerated degradation due to external environmental and internal physiological influences. The resistant variety was more adaptable and, thus, had higher chlorophyll contents than the sensitive variety on both Days 5 and 14. Because high RH indirectly limits nutrient uptake from the soil, reduced transpiration may result in increased soil water content, leading to changes in root morphology and ectomycorrhizal communities, leading to reduced phosphorus uptake, all of which may result in a decrease in chlorophyll content [13,66]. The increase in the MDA content observed may, in turn, have been due to increases in reactive oxygen species and cell membrane peroxidation with increasing stress duration and damage accumulation, which leads to the failure of normal plant growth [67]. In cucumber, low temperature and high RH stress was shown to reduce plant growth, photosynthesis, and chlorophyll biosynthesis, in addition to causing changes in hormone profiles, upregulation of antioxidants, reduced MDA, and increased reactive oxygen accumulation [6]. Our findings do not agree with those of previous studies. The MDA levels were significantly higher in the treatment groups than in the control groups, but the resistant variety had lower levels than the sensitive variety, indicating less damage. Of the three indicators of enzyme activity, two showed higher activity in the resistant variety than in the sensitive variety, aiding in the scavenging of reactive oxygen species produced as a result of the HR stress [68], thus reducing the damage. Most notably, ABA, which significantly increases in response to most adaptive stresses, such as drought and cold stress, to ensure plant adaption [69], was significantly downregulated after RH treatment, and the ABA levels were significantly lower in the sensitive variety than in the resistant variety. Growing evidence indicates that incubation under high RH not only reduces stomatal responsiveness, but also reduces ABA contents [38,39,40]. This is related to the fact that high RH is generally accompanied by a higher leaf water potential [28]. It has been suggested that, in varieties in which stomatal function is barely affected by high RH, the ABA content decreases only slightly, whereas in varieties in which high RH causes a significant attenuation of stomatal function, the ABA content decreases significantly [11].

Metabolomic analysis revealed that metabolites were strongly upregulated in the sensitive variety after 5 days of stress, and most of these were lipids and flavonoids. In contrast, the phenolic acids and flavonoids of the highly resistant variety were only detected in significant quantities after 14 days of stress. This, combined with the morphological and physiological indicators, gives us reason to suspect that the sensitive variety entered prematurely into a stress response because of its poor adaptability, as flavonoids as stressors not only accumulate rapidly when plants are damaged, their accumulation inhibits growth hormone transport and, thus, reduces growth [70,71]. These findings also explain the significantly better growth of the highly resistant material than the sensitive material upon 5 days of high RH. In addition, we found that arginine biosynthesis was significantly enriched in the highly resistant material regardless of stress duration. Arginine is known to be a precursor of NO and can be converted to polyamines, which both can play key regulatory roles during development and in responses to abiotic stresses. Moreover, arginine is also a major storage and transport form of organic nitrogen in plants [72].

Combining the transcriptome and metabolome data, we found that plant hormone signaling pathways were significantly altered in response to high RH stress, with auxin, ABA, and JA being particularly responsive. Given their importance in abiotic stresses [73], we further validated our findings by analyzing key genes related to growth factors, namely related to ABA and JA signaling. JA-Ile is a key signaling molecule that activates JA-mediated developmental and injury stress responses, whereas JAR1 is a JA-monoacyl amino acid synthetase that binds JA to isoleucine. In this study, the JAR1-related gene LOC110725177 was significantly upregulated in the treatment group, which in general agrees with the results of previous studies, since JA has a prominent role in managing stress [74,75,76]. As for ABA, we validated the ABA-related genes LOC110717340 and LOC110710716, which are homologs of AtNCED (9-cis-epoxycarotenoid dioxygenase) and CYP707A (ABA 8′-hydroxylase), respectively. In a previous study, the mutation of these genes resulted in reduced ABA levels, which play an important physiological role in the response to high RH [77]. In line herewith, the two genes were significantly downregulated in both treatment groups in our study. Therefore, we suggest that, in the face of high RH stress, quinoa seedlings reduce endogenous ABA, increase the JA content, and upregulate auxin synthesis to regulate the expression of downstream stress response genes. Moreover, correlation analyses also showed that pem1651 (IAA) and pem1654 (JA) were highly correlated with most genes after high humidity treatments and that the correlation between pem1651 and the differential genes was significantly higher in resistant than in sensitive materials.

Of all the transcription factors we identified, the most abundant in the high-resistance material was FAR1, whereas AP2\ERF was the most-highly expressed transcription factor in the treatment groups compared with controls. FAR1 is known to play a role in multiple developmental and physiological processes, including ultraviolet-B signaling, biological clock rhythms, flowering, chloroplast biogenesis, chlorophyll biosynthesis, programmed cell death, reactive oxygen species homeostasis, and ABA signaling [78]. Similarly, AP2/ERF is a major regulator of various stress responses and can also respond to hormonal regulation, including ABA- and ethylene-mediated signals [79,80]. ABA, in particular, has an important role in regulating transcription factor activity in response to stress [81]. Since the present study was conducted in a greenhouse in which the light patterns did not fully mimic the natural environment, the response of the FAR1 transcription factor could also affect the starch synthesis and metabolism in a light-dependent manner [82]. We also screened five possible long-lasting response genes in the resistant materials based on combined WGCNA and PPI analyses. Among them were identified LOC110722395 (phosphoglycerate kinase, chloroplastic), LOC110733932 (chlorophyll synthase, chloroplastic), and LOC110736961 (E3 ubiquitin-protein ligase), which are associated with chloroplasts [83]; LOC110706418 (serine hydroxymethyltransferase, mitochondrial), which is associated with mitochondria; and LOC110718123 (heat shock factor protein HSF24), which is associated with stress. Hence, these longevity genes in highly resistant materials are associated with photosynthesis, energy metabolism, and stress response. WGCNA data on morphological and physiological indicators, along with all the identified genes, also showed that most of the candidate genes that were highly correlated with morphology encoded stress-, heat response-, and calcium-related proteins. Among the candidate genes highly correlated with physiology were genes related to basic cell wall components and the ABA receptor. All of the above-described features may help understand why highly resistant materials have better growth patterns under high RH stress. Moreover, we believe that the response to high RH stress is also influenced by the external environment and other intrinsic metabolic pathways, not just single hormone signaling pathways. This will be our focus in future studies on RH in quinoa.

## 5. Conclusions

In conclusion, this study revealed significant changes in morphological and physiological indicators in seedlings of both resistant and sensitive quinoa varieties under high RH treatment. Combined transcriptome and metabolome data analysis revealed the response mechanisms of DEGs and DAMs in the resistant variety to high RH stress. We confirmed the response patterns of ABA-related genes LOC110717340 and LOC110710716, the auxin-related gene LOC110706251, and the JA-related gene LOC110725177 in response to high RH. We identified genes and metabolites that may be involved in the regulation of high RH responses, most of which involve stress-, heat shock-, calcium-associated proteins and cell-wall-component-associated and ABA receptor genes. Regulatory network analysis revealed hub genes related to hormone signaling, suggesting that these genes play an important role in the response to high RH. Future studies of these genes and interaction networks may shed new light on the response of quinoa to high RH.

## Figures and Tables

**Table 1:** Details of sample numbers.

Materials	Features	Treatment Modalities	Time/Name ^1^
Dianli-969	Sensitive type	Normothermic (25 ± 1 °C); High humidity (90 ± 2%)	5d/TS1; 14d/TS2
		Normothermic (25 ± 1 °C); Normal humidity (50 ± 2%)	5d/CS1; 14d/CS2
Dianli-439	Highly resistant	Normothermic (25 ± 1 °C); High humidity (90 ± 2%)	5d/TR1; 14d/TR2
		Normothermic (25 ± 1 °C); Normal humidity (50 ± 2%)	5d/CR1; 14d/CR2

^1^ 5d/TS1, sensitive-type materials in high humidity stress for 5 days; 5d/CS1, control of sensitive-type materials in high humidity stress for 5 days; 14d/TS2, sensitive-type materials in high humidity stress for 14 days; 14d/CS2, control of sensitive-type materials in high humidity stress for 14 days; 5d/TR1, highly resistant materials in high humidity stress for 5 days; 5d/CR1, control of highly resistant material in high humidity stress for 5 days; 14d/TR2, highly resistant materials in high humidity stress for 14 days; 14d/CR2, control of highly resistant materials in high humidity stress for 14 days.

**Table 2:** Changes in morphological indicators for the two varieties after different periods of stress.

Processing Time (Days)	Group Name	Plant Height (cm)	Leaf Area (mm^2^)	Fresh Weight (g)	Dry Weight (g)	Root-to-Crown Ratio
5	CR1	7.83 ± 0.28 ^a^	281.93 ± 22.81 ^a^	0.4292 ± 0.0329 ^a^	0.0602 ± 0.0075 ^a^	0.0429 ± 0.0022 ^a^
	TR1	6.91 ± 0.86 ^a^	271.54 ± 22.78 ^a^	0.3772 ± 0.0287 ^a^	0.0190 ± 0.0051 ^b^	0.0490 ± 0.006 ^a^
	CS1	7.77 ± 1.04 ^a^	274.69 ± 17.62 ^a^	0.4133 ± 0.0387 ^a^	0.0548 ± 0.0048 ^a^	0.0445 ± 0.0067 ^a^
	TS1	6.89 ± 0.98 ^a^	267.57 ± 28.52 ^a^	0.3818 ± 0.0638 ^a^	0.0201 ± 0.0109 ^b^	0.050 ± 0.0008 ^a^
14	CR2	11.51 ± 1.37 ^a^	559.65 ± 34.9 ^a^	0.8699 ± 0.06816 ^a^	0.1214 ± 0.0071 ^a^	0.084 ± 0.0234 ^b^
	TR2	10.89 ± 1.16 ^a^	484.44 ± 92.78 ^a^	0.7349 ± 0.1530 ^a^	0.0310 ± 0.0069 ^a^	0.099 ± 0.0137 ^a^
	CS2	11.45 ± 0.88 ^a^	549.67 ± 70.60 ^a^	0.8785 ± 0.1039 ^a^	0.1196 ± 0.0157 ^a^	0.100 ± 0.0164 ^a^
	TS2	8.38 ± 1.50 ^b^	421.98 ± 35.12 ^b^	0.5901 ± 0.0557 ^b^	0.0236 ± 0.0069 ^b^	0.067 ± 0.0037 ^b^

Data presented as the mean ± SE (*n* = 3). Values with matching letters are not significantly different from each other (*p* > 0.05).

**Table 3:** Changes in physiological indicators in the two varieties after different periods of stress.

Time (Days)	Group Name	CHL (μg·mL^−1^)	ABA (ng·g^−1^)	SOD (U·mg^−1^ FW)	POD (U·mg^−1^ FW)	CAT (U·mg^−1^ FW)	MDA (μmol·g^−1^ FW)
5	CR1	37.26 ± 3.985 ^a^	73.10 ± 16.29 ^a^	99.28 ± 9.215 ^c^	1217.8 ± 128.3 ^c^	3133.0 ± 151.9 ^b^	8.056 ± 1.3045 ^c^
	TR1	30.48 ± 5.427 ^b^	54.03 ± 5.635 ^b^	315.89 ± 29.68 ^a^	2253.3 ± 242.8 ^b^	4099.5 ± 102.7 ^a^	17.48 ± 2.3631 ^b^
	CS1	38.78 ± 6.916 ^a^	76.71 ± 8.499 ^a^	108.11 ± 13.45 ^c^	1111.6 ± 128.1 ^c^	2973.6 ± 49.9 ^b^	9.894 ± 1.8532 ^c^
	TS1	21.53 ± 2.064 ^b^	53.69 ± 5.374 ^b^	244.15 ± 44.57 ^b^	2536.1 ± 156.9 ^a^	3289.8 ± 252.2 ^b^	25.49 ± 3.7486 ^a^
14	CR2	40.74 ± 5.201 ^a^	84.23 ± 4.176 ^b^	125.31 ± 19.45 ^c^	1446.6 ± 134.4 ^c^	3256.5 ± 212.6 ^c^	8.563 ± 2.5638 ^c^
	TR2	27.79 ± 3.165 ^b^	65.14 ± 22.71 ^c^	500.93 ± 35.13 ^a^	2241.2 ± 172.2 ^b^	4756.2 ± 118.4 ^a^	25.64 ± 5.383 ^b^
	CS2	38.91 ± 5.799 ^a^	93.83 ± 2.724 ^a^	82.56 ± 6.314 ^d^	1341.5 ± 122.8 ^c^	3300.9 ± 173.7 ^c^	7.478 ± 1.8581 ^c^
	TS2	19.96 ± 3.931 ^c^	41.1 ± 12.10 ^d^	434.71 ± 16.54 ^b^	3132.9 ± 224.0 ^a^	4060.8 ± 565.5 ^b^	37.72 ± 4.861 ^a^

Data presented as the mean ± SE. Values with matching letters are not significantly different from each other (*p* > 0.05).

**Table 4:** Differentially expressed transcription factors under high RH stress.

Gene Family	Number of DEGs	DEGs Description	Biological Functions
FAR1	566	Far-Red-Impairedresponse1	Development and stress response
AP2/ERF	130	APETALA 2/ethylene-responsive element binding factor	Development and stress response
bHLH	202	Basic helix-loop-helix TFs	Cell development and substance metabolism
C2H2	127	C2H2 zinc finger protein	Development and stress response
C3H	94	C3H zinc finger protein	Embryo formation
bZIP	94	box-zipper protein	Plant photomorphogenesis and fruit ripening
MYB	147	MYB TFs	Cell development and flavonoid pathway
NAC	115	NAC family TFs	Development and stress response
WRKY	102	WRKY DNA-binding protein	Defense responses and plant development
B3	155	B3-domain-containing protein	Plant growth and development
Others	1210		
Total	2825		

Differentially expressed genes were identified by FDR ≤ 0.05 and an absolute value of log2ratio ≥ 1.

**Table 5:** Gene function in important module candidate genes.

Modules	Module Candidate Hub Genes	Hub Genes’ Description
Black	LOC110729592	Belongs to the sterol desaturase family
	LOC110716937	Beta-D-xylosidase (BXL3)
	LOC110724693	Belongs to the cytochrome P450 family (CYP83A1)
	LOC110736400	Calreticulin-3-like
	LOC110683032	Belongs to the protein kinase superfamily
	LOC110701145	Wall-associated receptor kinase-like
	LOC110712611	Glycosyl transferase family 17 protein
	LOC110706702	MADS-box protein
	LOC110701071	Calreticulin-3-like
	LOC110716012	Glucose and ribitol
	LOC110715868	Sucrose-cleaving enzyme that provides UDP-glucose and fructose for various Metabolic pathways (SUS1)
	LOC110733529	Amino acid transporter
	LOC110695772	\
	LOC110708285	Ammonium transporter (AMT3-1)
	LOC110717076	Lob-domain-containing protein
	LOC110721833	Sucrose-cleaving enzyme that provides UDP-glucose and fructose for various metabolic pathways (SUS1)
	LOC110709750	MADS-box protein
	LOC110728040	Cystinosin homolog
	LOC110686969	Protein plant cadmium resistance
	LOC110698492	Partial alpha-/beta-hydrolase lipase region
	LOC110684083	Belongs to the heat shock protein 70 family
	LOC110682201	Major facilitator superfamily protein
	LOC110706950	Serine threonine-protein kinase
	LOC110733057	Universal stress protein
	LOC110687281	Cysteine-rich receptor-like protein kinase
	LOC110729587	Heat shock protein
	LOC110696285	Transposition, RNA-mediated
Blue	LOC110738464	Transporter
	LOC110705966	Glycerophosphoryl diester phosphodiesterase
	LOC110731861	Alcohol dehydrogenase GroES-like domain
	LOC110688479	Protein upstream of
	LOC110723960	Glycerophosphoryl diester phosphodiesterase
	LOC110710755	Abscisic acid receptor
	LOC110732716	Zinc transporter
	LOC110686655	Polyamine oxidase
	LOC110723200	Belongs to the chalcone isomerase family
	LOC110718261	Rho GTPase-activating protein
	LOC110695938	Belongs to the Casparian strip membrane proteins (CASP) family
	LOC110685067	Oligopeptide transporter
	LOC110700433	Belongs to the cytochrome P450 family
	LOC110690627	Belongs to the glycosyl hydrolase 5 (cellulase A) family
	LOC110723446	Belongs to the protein kinase superfamily (SAPK2)
	LOC110735654	Choline kinase
	LOC110709626	Catalyzes xyloglucan endohydrolysis (XEH) and or endotransglycosylation (XET); cleaves and relegates xyloglucan polymers, an essential constituent of the primary cell wall, and thereby participates in cell wall construction of growing tissues
	LOC110704465	Atexp8, atexpa8, athexp alpha 1.11, exp8, expa8 (EXP2)
	LOC110733886	Abscisic acid receptor
	LOC110736716	calcium-dependent protein kinase (CDPK9)
	LOC110710508	GAST1-like
	LOC110691154	phosphoenolpyruvate carboxylase
	LOC110739071	Protein NRT1 PTR FAMILY

## Data Availability

The original contributions presented in the study are publicly available. These data can be found in the National Center for Biotechnology Information (NCBI) SRA database under Accession Number PRJNA953156.

## Supplementary Materials

The following supporting information can be downloaded at: https://www.mdpi.com/article/10.3390/biom13091352/s1.
