# Supplementary material for: Integrated Metabolomic and Transcriptomic Analysis of the Quinoa Seedling Response to High Relative Humidity Stress
Source: Biomolecules. 2023 Sep 5;13(9):1352. doi: 10.3390/biom13091352 (PMC10527060; doi:10.3390/biom13091352)
Supplement: Supplementary file 1 [file biomolecules-13-01352-s001.zip › biomolecules-2566255-supplementary.pdf]

(A)

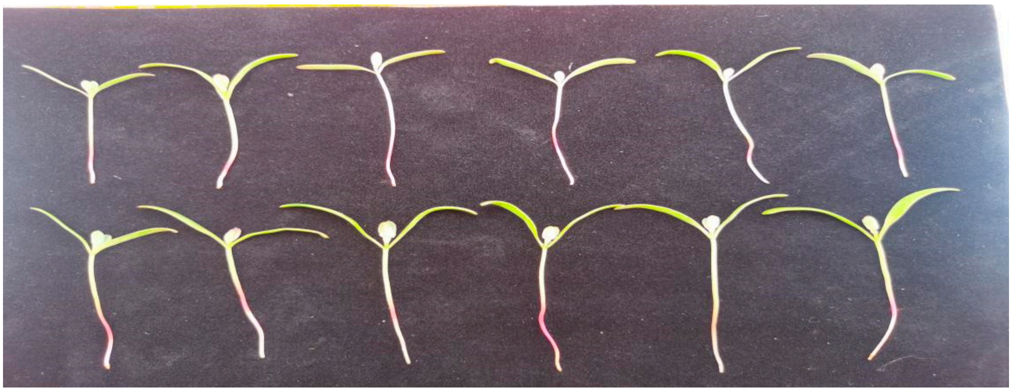

(B)

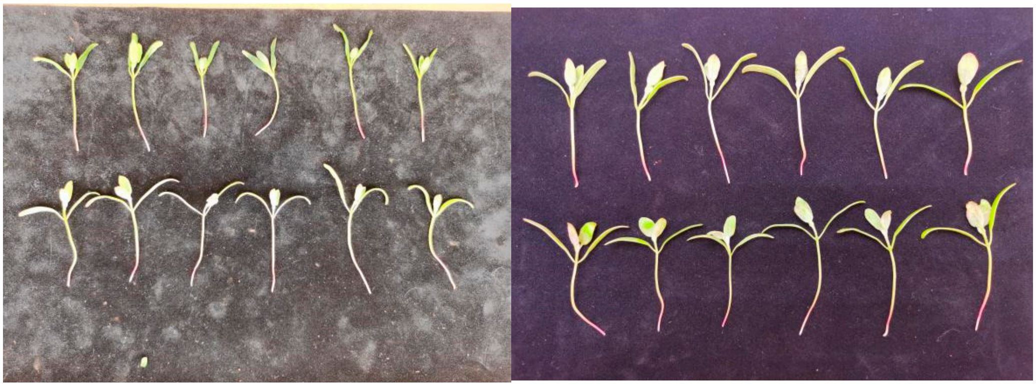

(C)

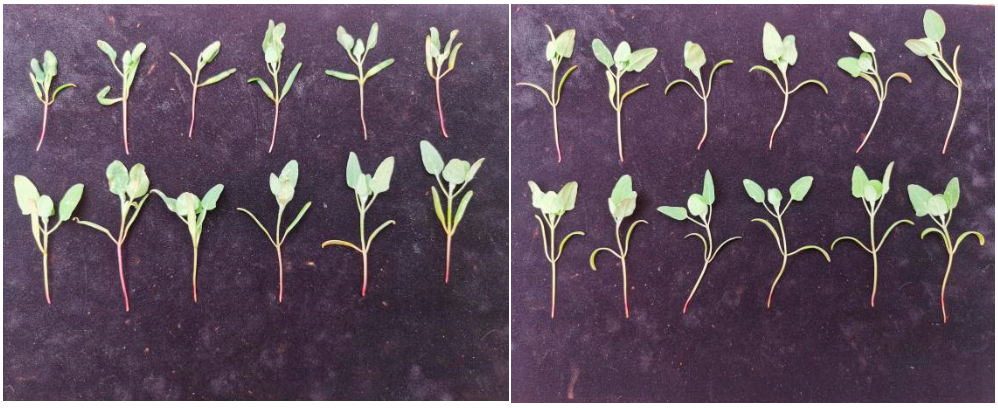

Figure S1. Morphology of sensitive (top) and highly resistant (bottom) seedling strains**.** (**A**) Before treatment. **(B)** After 5 days of treatment. **(C)** After 14 days of treatment.

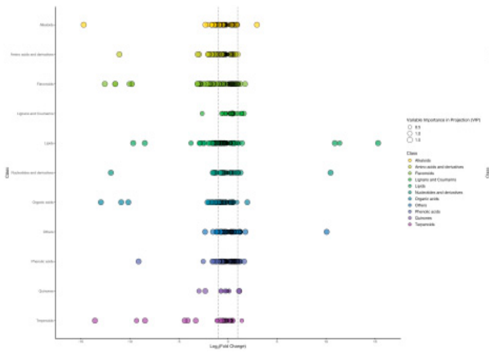

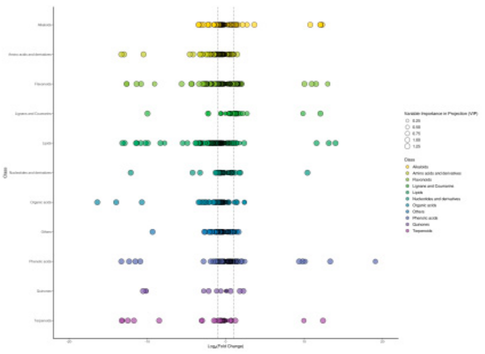

TR1 vs. CR1

TR2 vs. CR2

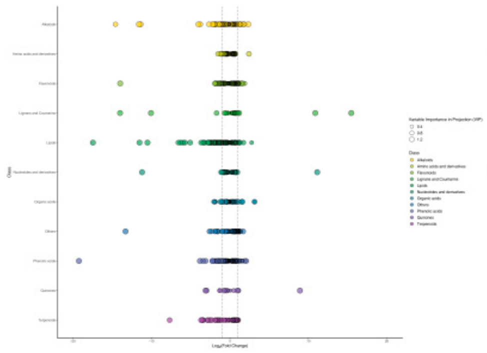

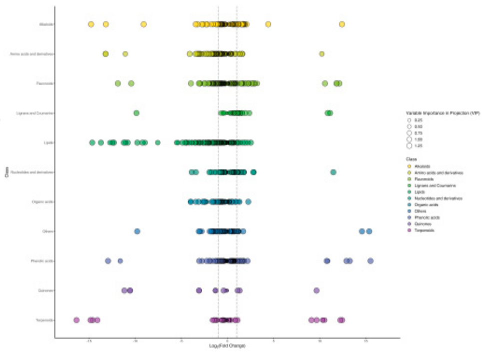

TS1 vs. CS1

TS2 vs. CS2

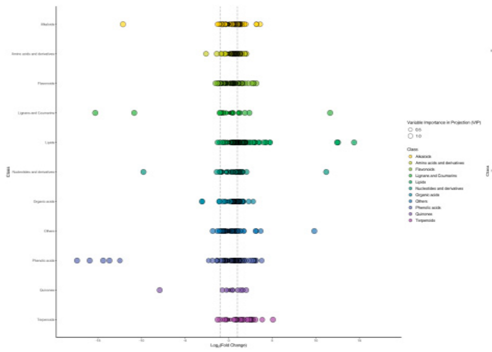

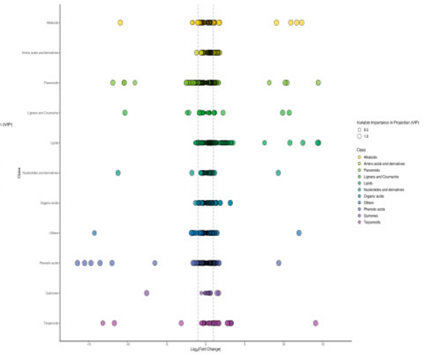

TR1 vs. TS1

TR2 vs. TS2

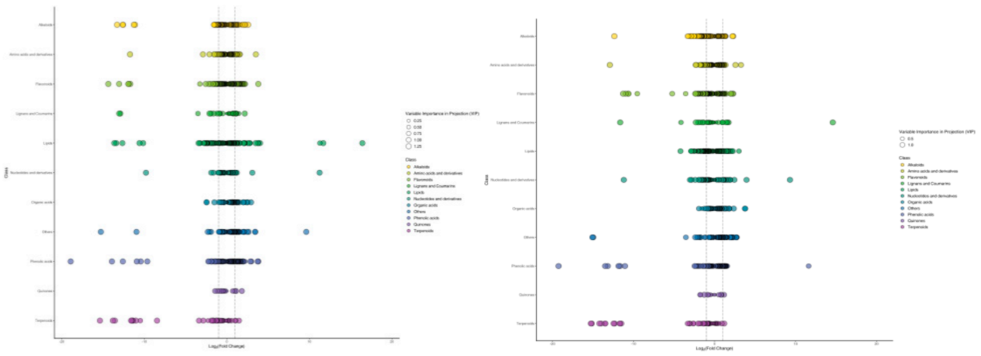

TR1 vs. TR2 TS1 vs. TS2

Figure S2. Identification of the differently accumulated metabolites (DAM).

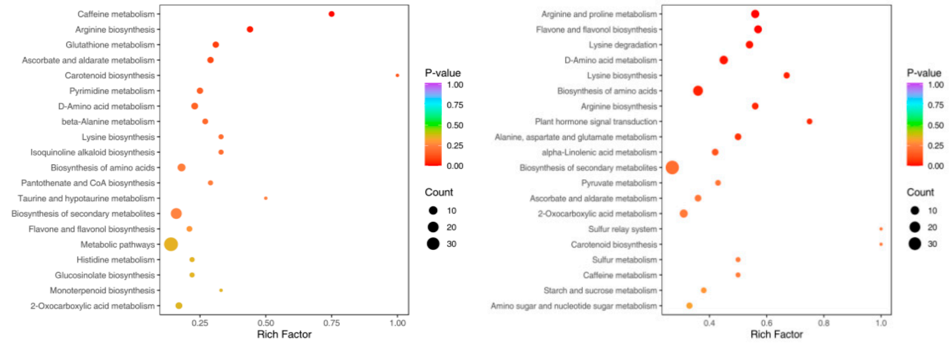

TR1 vs. CR1 TR2 vs. CR2

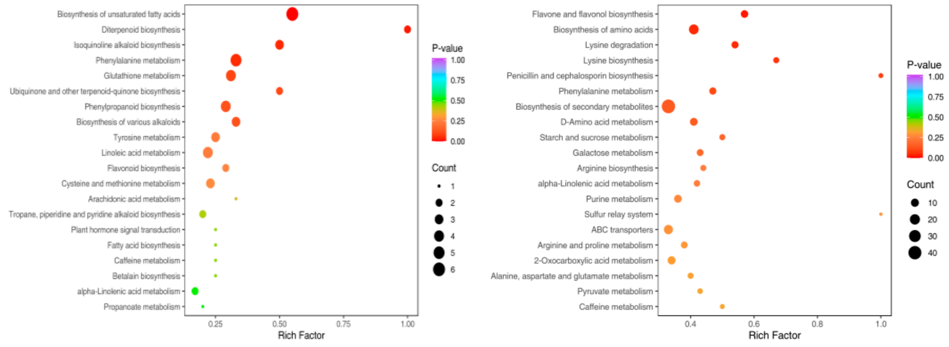

TS1 vs. CS1 TS2 vs. CS2

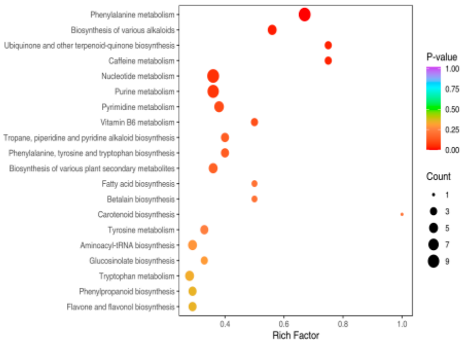

TR1 vs. TS1

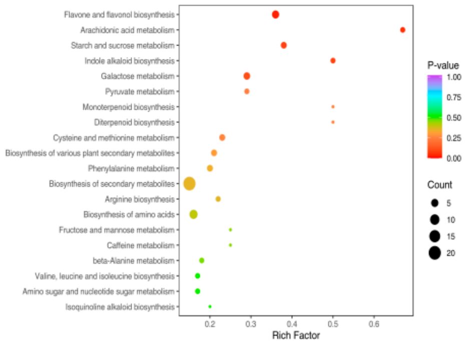

TR2 vs. TS2

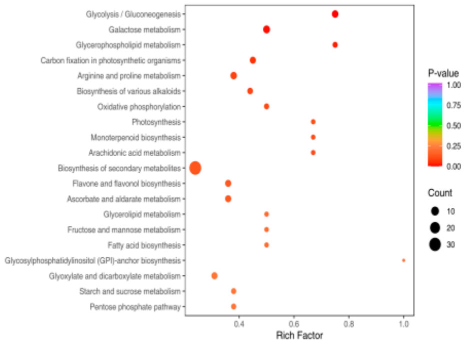

TR1 vs. TR2

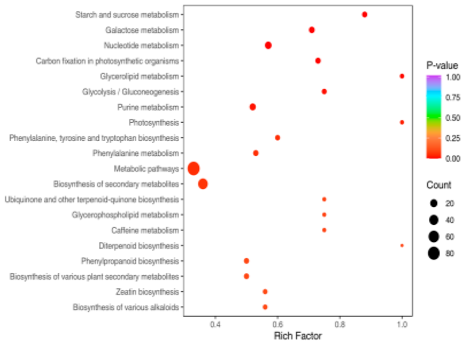

TS1 vs. TS2

Figure S3. KEGG pathway enrichment analysis of the DAM.

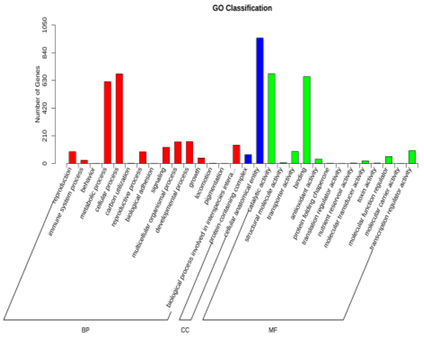

TR1 vs. CR1

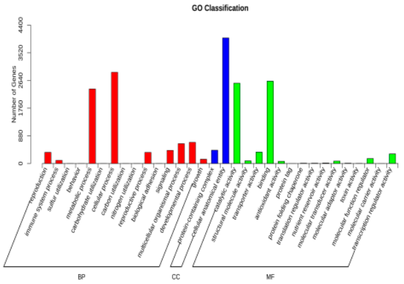

TR2 vs.CR2

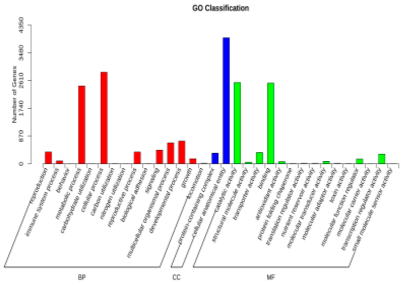

TS1 vs.CS1

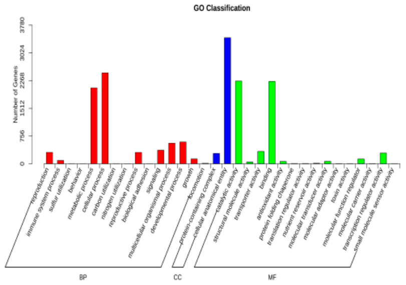

TS2 vs.CS2

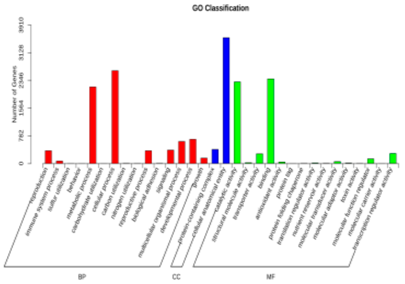

TR1 vs.TS1

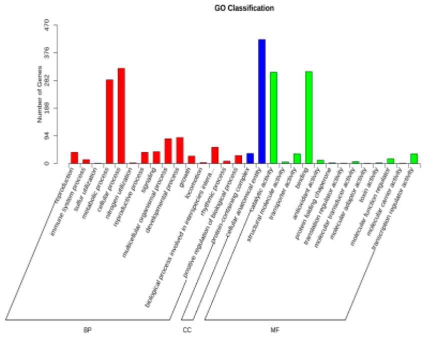

TR2 vs.TS2

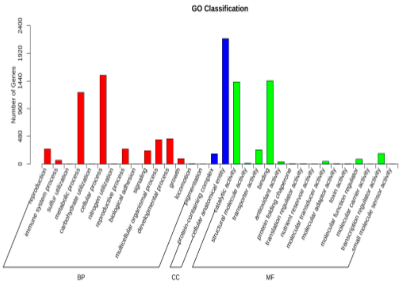

TR1 vs.TR2

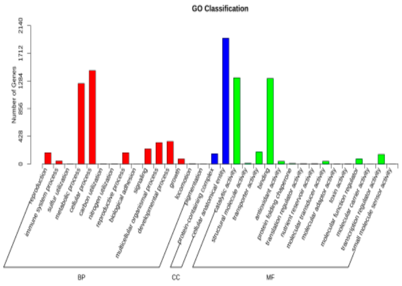

TS1 vs.TS2

Figure S4. GO enrichment analysis of the DEGs.

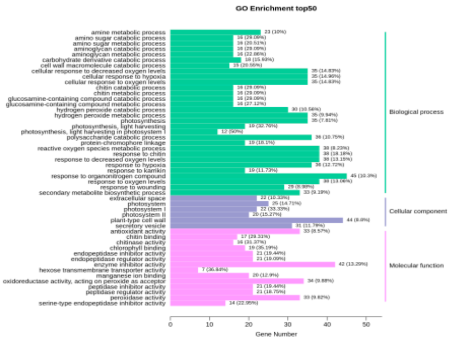

TR1 vs. CR1

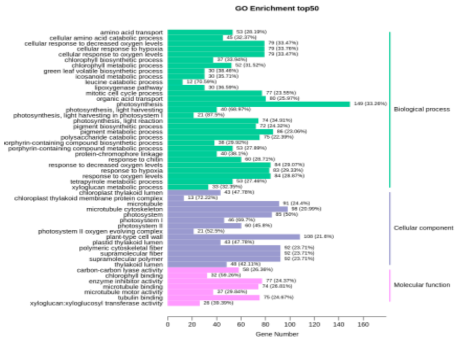

TR2 vs. CR2

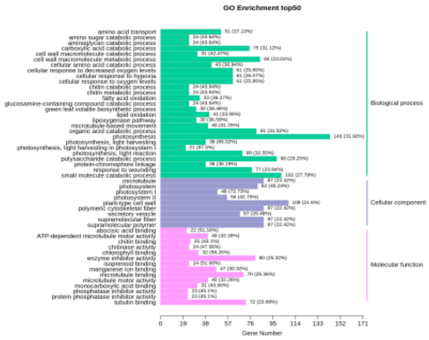

TS1 vs. CS1

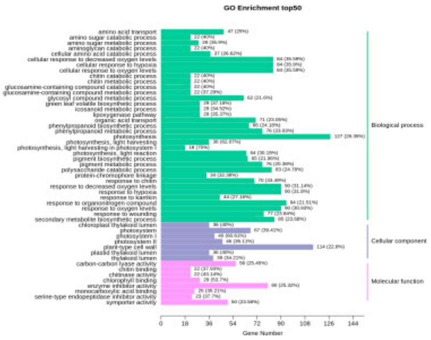

TS2 vs. CS2

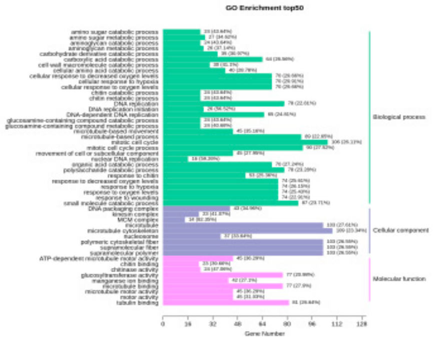

TR1 vs. TS1

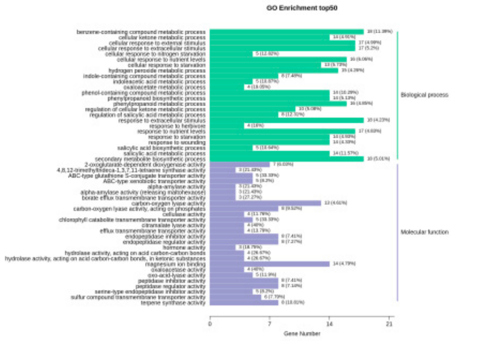

TR2 vs. TS2

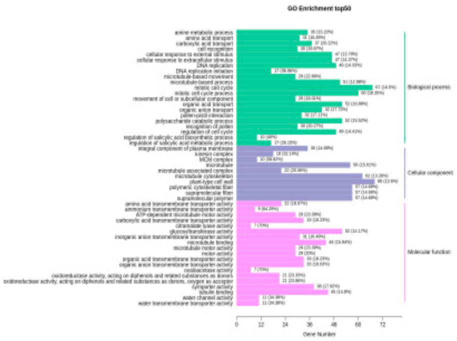

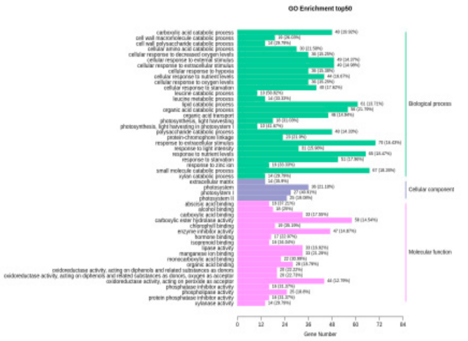

TR1 vs. TR2

TS1 vs. TS2

Figure S5. Top 50 enriched biological processes in each group of DEGs.

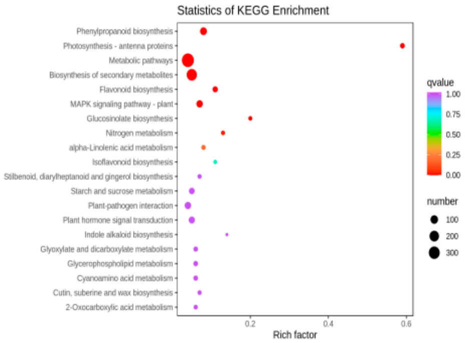

TR1 vs. CR1

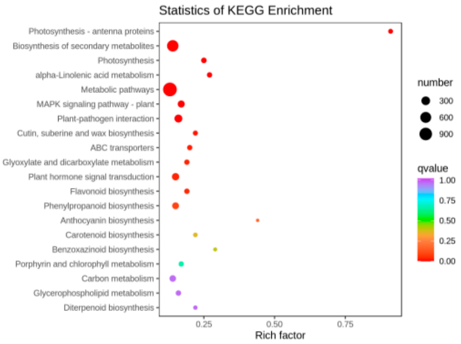

TR2 vs. CR2

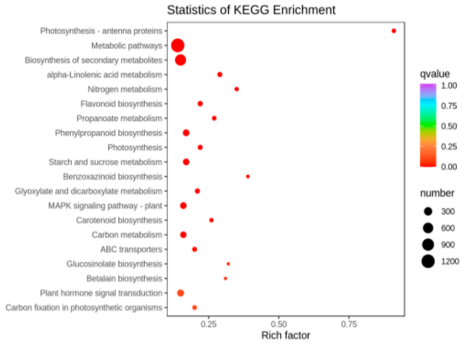

TS1 vs. CS1

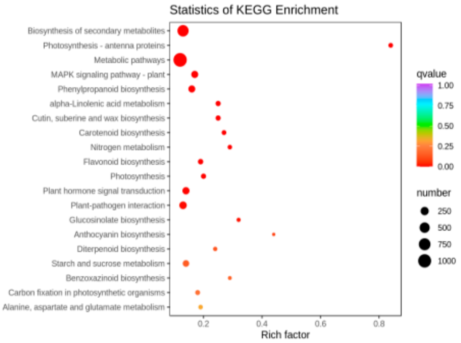

TS2 vs. CS2

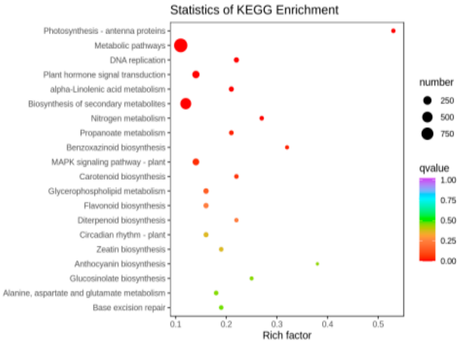

TR1 vs. TS1

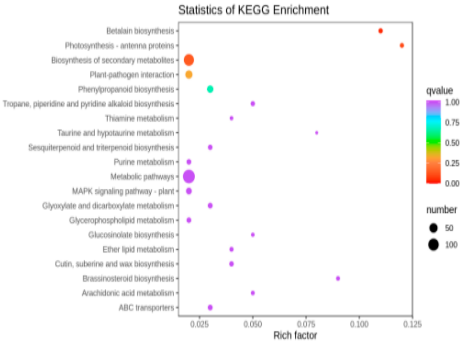

TR2 vs. TS2

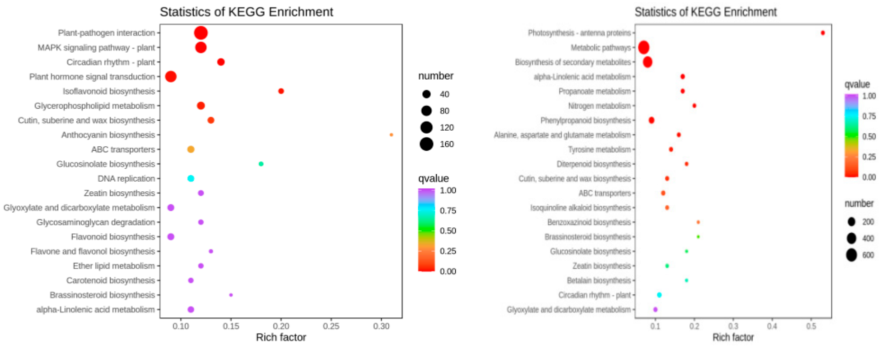

TR1 vs. TR2 TS1 vs. TS2

Figure S6. KEGG pathway enrichment analysis of the DEGs.

A

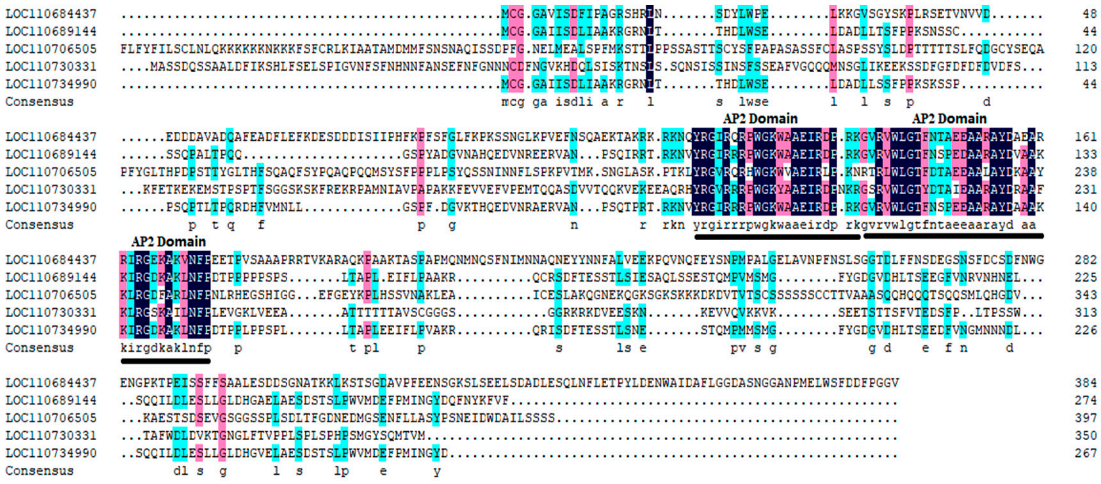

B

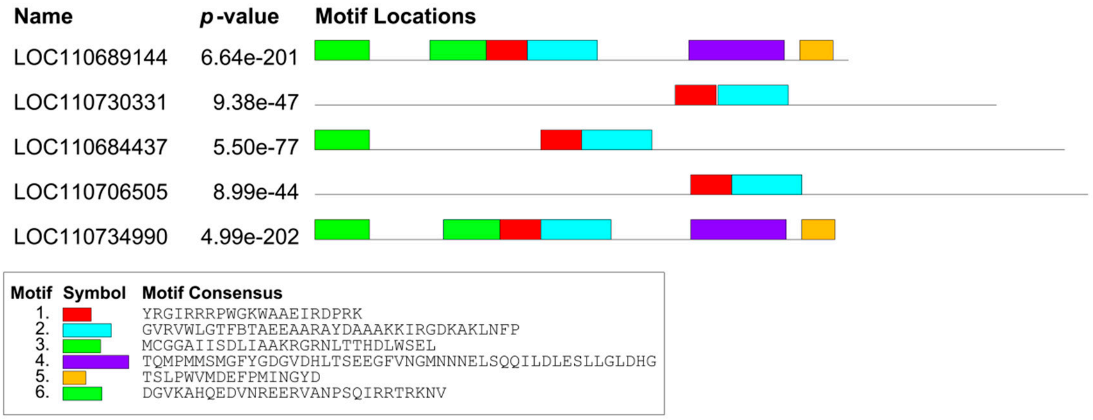

C

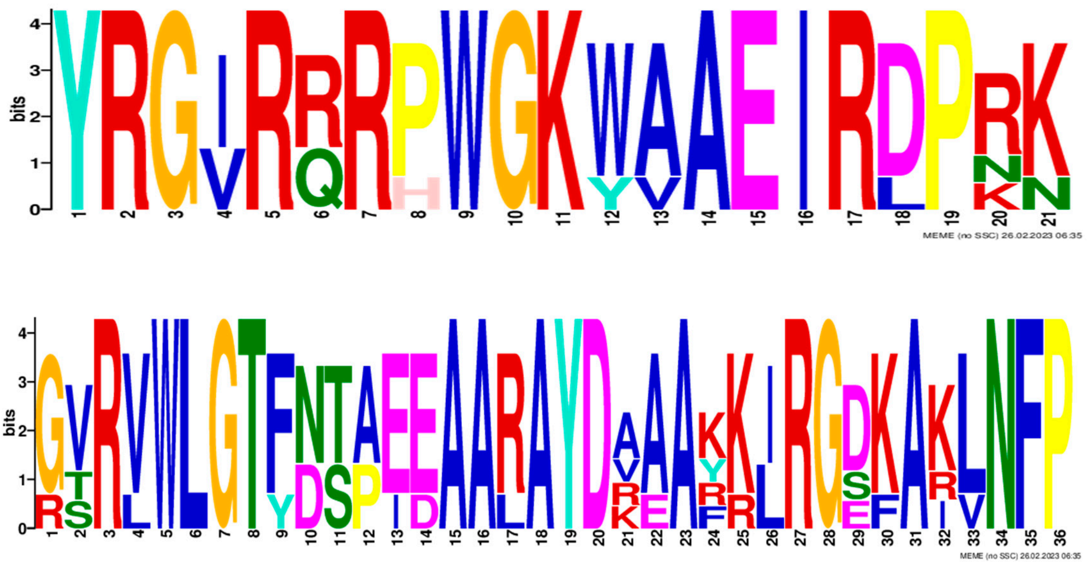

Figure S7. Comparative analysis of five AP2/ERF family genes. **(A)** multiple sequence alignment. **(B)** structural domain analysis. **(C)** conserved structural domain sequence tags.

A

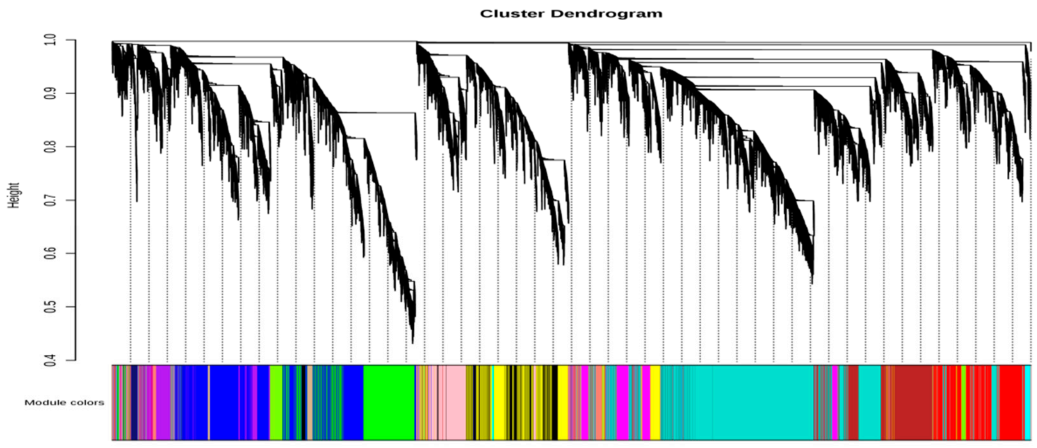

B

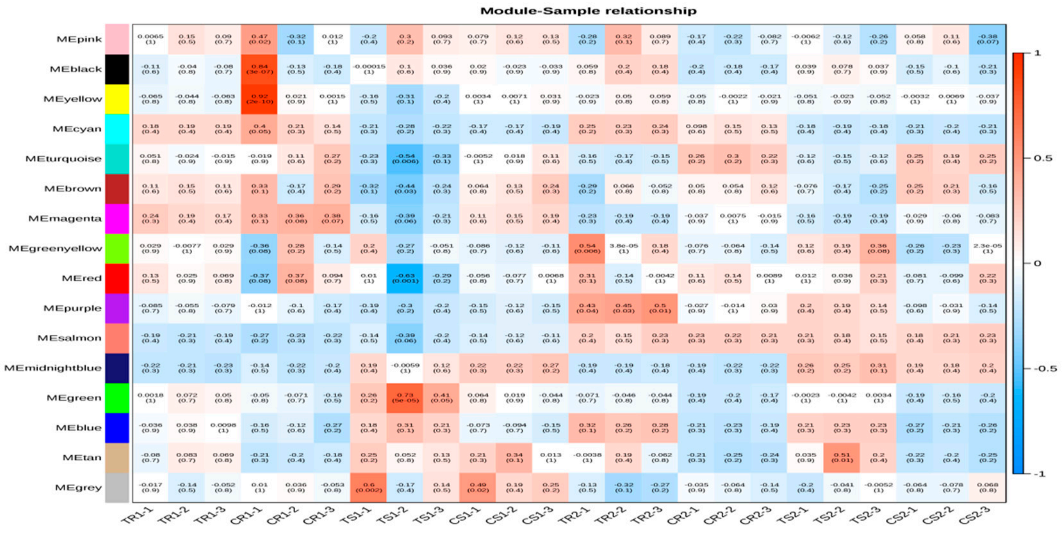

Figure S8. Weighted correlation network analysis of the DEGs. **(A)** Clustering and module identification of the DEGs. Each row represents a color-coded module containing a set of highly linked genes. **(B)** Heatmap of the gene co-expression network modules in each sample. Module names and time points are shown on the y-axis and x-axis, respectively. Red and blue colors represent positive and negative correlations, respectively, according to the indicated legend.
